# Roquin binds microRNA-146a and Argonaute2 to regulate microRNA homeostasis

**DOI:** 10.1038/ncomms7253

**Published:** 2015-02-20

**Authors:** Monika Srivastava, Guowen Duan, Nadia J. Kershaw, Vicki Athanasopoulos, Janet H. C. Yeo, Toyoyuki Ose, Desheng Hu, Simon H. J. Brown, Slobodan Jergic, Hardip R. Patel, Alvin Pratama, Sashika Richards, Anil Verma, E. Yvonne Jones, Vigo Heissmeyer, Thomas Preiss, Nicholas E. Dixon, Mark M. W. Chong, Jeffrey J. Babon, Carola G. Vinuesa

**Affiliations:** 1 Department of Pathogens and Immunity, John Curtin School of Medical Research, Canberra, Australian Capital Territory 2601, Australia; 2 Division of Structural Biology, Walter and Eliza Hall Institute and The University of Melbourne, Melbourne, Victoria 3052, Australia; 3 Genomics and Immunology laboratory, St Vincent’s Institute of Medical Research, Fitzroy, Victoria 3065, Australia; 4 Wellcome Trust Centre for Human Genetics, University of Oxford, Roosevelt Drive, Oxford OX3 7BN, UK; 5 Helmholtz Zentrum München, Institute of Molecular Immunology, D-81377 München, Germany; 6 Centre for Medical and Molecular Bioscience, University of Wollongong and Illawarra Health and Medical Research Institute, Wollongong, New South Wales 2522, Australia; 7 Department of Genome Biology, John Curtin School of Medical Research, Canberra, Australian Capital Territory 2601, Australia; 8 Genome Discovery Unit, John Curtin School of Medical Research, Canberra, Australian Capital Territory 2601, Australia; 9 Ludwig-Maximilians-Universität München, Institute for Immunology, D-80336 München, Germany; 10 Victor Chang Cardiac Research Institute, Darlinghurst, New South Wales 2010, Australia

## Abstract

Roquin is an RNA-binding protein that prevents autoimmunity and inflammation via repression of bound target mRNAs such as inducible costimulator (*Icos*). When Roquin is absent or mutated (Roquin^san^), *Icos* is overexpressed in T cells. Here we show that Roquin enhances Dicer-mediated processing of pre-miR-146a. Roquin also directly binds Argonaute2, a central component of the RNA-induced silencing complex, and miR-146a, a microRNA that targets *Icos* mRNA. In the absence of functional Roquin, miR-146a accumulates in T cells. Its accumulation is not due to increased transcription or processing, rather due to enhanced stability of mature miR-146a. This is associated with decreased 3′ end uridylation of the miRNA. Crystallographic studies reveal that Roquin contains a unique HEPN domain and identify the structural basis of the ‘*san’* mutation and Roquin’s ability to bind multiple RNAs. Roquin emerges as a protein that can bind Ago2, miRNAs and target mRNAs, to control homeostasis of both RNA species.

## Abstract (web summary)

Roquin is an RNA-binding protein that promotes the degradation of specific mRNAs and is crucial for the maintenance of peripheral immune tolerance. Here the authors show that, in addition to its target mRNAs, Roquin can bind miR-146a and the RISC component Ago2 to control homeostasis of both RNA species.

## Graphical abstract

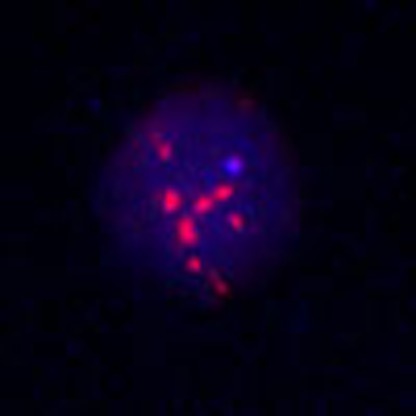

Posttranscriptional gene regulation by *trans*-acting RNA-binding proteins is a rapid and efficient way to modify gene expression and cellular responses. Mutations in these *trans*-acting factors are often associated with pathology^1^. The protein Roquin (encoded by *Rc3h1*), known to bind target mRNAs and promote their decay, has been found to be crucial in the maintenance of peripheral immune tolerance. Mice either lacking Roquin/Roquin2 or homozygous for the Roquin *‘san’* mutation (*sanroque* mice) have impaired posttranscriptional regulation of mRNAs in T cells and macrophages^2^,^3^,^4^,^5^,^6^. Roquin is a ubiquitously expressed RING-E3 ubiquitin ligase family member containing a highly conserved ROQ domain required for RNA binding and localization to stress granules^7^,^8^ and a CCCH zinc finger motif. Roquin binds a stem-loop structure termed the constitutive decay element (CDE) within the 3′-UTR of *Tnf* mRNA and recruits the Ccr4-Caf1-Not deadenylation machinery leading to mRNA decay^6^. mRNA degradation can also be mediated by Roquin’s recruitment of Rck/Edc4/Dcp1a decapping complexes and is thought to occur independently of miRNA activity^8^.

MicroRNAs (miRNAs) are small 20−22 nt non-coding RNAs that regulate specific mRNA targets mainly by translational inhibition and/or mRNA decay^9^. They are involved in most cellular responses and their dysregulation has been shown to be associated with autoimmune diseases^10^. Synthesized in the nucleus as long primary transcripts, they are cleaved into precursor miRNAs (pre-miRNAs) and exported to the cytoplasm where the hairpin structure is processed by the RNaseIII complex Dicer/Tarbp2 into the mature ~22 nt long imperfect miRNA duplex. This is loaded into the miRNA RNA-induced silencing complex (miRISC), comprised of several proteins including GW182 and Ago2. Once loaded into the miRISC, miRNAs interact with sequences within target mRNAs^9^,^11^.

Although much is known about miRNA biogenesis and function, relatively little is known about miRNA homeostasis and its importance in immunity or autoimmunity. Specific nucleotides present in mature miRNAs, terminal-end modifications of miRNAs like adenylation or uridylation and subcellular localization have been shown to affect miRNA homeostasis but none of these mechanisms has yet been shown to regulate miRNA longevity in mammals^12^. Ribonucleases such as Sdn1 and Xrn1 regulate miRNA half-life in *Arabidopsis* and *Caenorhabditis elegans*, respectively^13^ but evidence of a similar role in mammalian cells is lacking. Recently Eri1, a highly conserved 3′-to-5′ exoribonuclease, was shown to regulate miRNA homeostasis in murine lymphocytes but the exact mechanism is still unknown^14^. Here we show that Roquin is one such regulator of miRNA longevity. Roquin binds to miR-146a and its target *Icos* mRNA and promotes their decay. Icos and miR-146a limit T follicular helper (Tfh) cells to prevent the development of autoantibodies and autoimmunity^15^. Roquin also forms a complex with the RISC component Ago2. These data implicate Roquin as a facilitator of miRNA-mediated target decay.

## Results

### Roquin represses miR-146a levels within T cells

**Figure 1:**
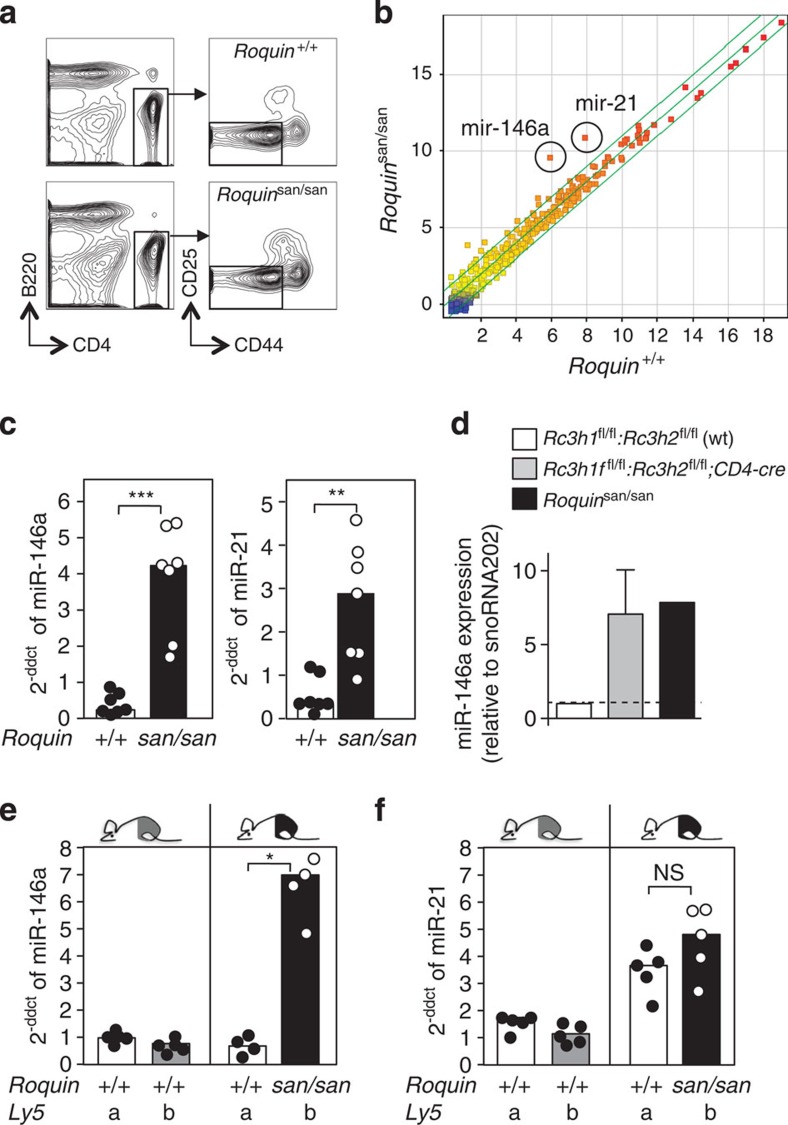
Loss of Roquin causes T cell-autonomous miRNA accumulation. (**a**) Flow cytometric stains from *Roquin*^san/san^ and *Roquin*^*+/+*^ T cells showing the gating strategy used to sort CD4^+^CD44^lo^CD25^−^ naive T cells. (**b**) Scatter plot of microRNA expression in sorted naive T cells obtained by microarray analysis. (**c**) qRT–PCR showing relative expression levels of mature miR-146a and miR-21 in naive T cells, normalized to U6. (**d**) Relative expression levels of miR-146a measured by qRT–PCR in *Rc3h1*^fl/fl^*;Rc3h2*^fl/fl^*; CD4-Cre* double knockout naive T cells normalized to snoRNA202. (**e**,**f**) qRT–PCR analysis of miR-146a (**e**) and miR-21 (**f**) in *Roquin*^san/san^.Ly5b: *Roquin*^*+/+*^.Ly5a or of *Roquin*^*+/+*^.Ly5b: *Roquin*^*+/+*^.Ly5a mixed chimeras. Each dot represents an individual mouse and the bar represents the median value in each group. *U*-test: *P<0.02, **P<0.05, ***P<0.005. See also Supplementary Fig. 1a,b.

We previously reported that several miRNAs were upregulated in T cells homozygous for the hypomorphic *‘san’* allele of *Roquin*^3^. Genome-wide miRNA profiling of purified naive CD4^+^ T cells (Fig. 1a) from *Roquin*^san/san^ and *Roquin*^*+/+*^ control littermates revealed 15 miRNAs were overexpressed by >twofold in *Roquin*^san/san^ T cells and none were downregulated (Fig. 1b and Supplementary Table 1). miR-146a (mmu-miR-146a-5p) and miR-21 (mmu-miR-21-5p) showed the greatest change: 25-fold and 14-fold, respectively. Quantitative real-time PCR (qRT–PCR) confirmed the increase in miRNA expression levels (Fig. 1c), which was not present in B cells, murine embryonic fibroblasts (MEFs) or other T-cell subsets (Supplementary Fig. 1a,b). Quantification of miR-146a in naive CD4^+^ T cells lacking both Roquin and Roquin-2 also revealed miR-146a accumulation (Fig. 1d) confirming that the failure of Roquin^san^ to limit miR-146a was a reflection of loss of function of the Roquin paralogues.

To test whether *Roquin*^san^ was acting within T cells to cause the increase in miR-146a, we constructed mixed bone marrow chimeric mice. For this, mice labelled with a congenic marker *(Ly5b*) were sublethally irradiated and reconstituted with a 50:50 mix of *Roquin*^san/san^.Ly5b and *Roquin*^*+/+*^.Ly5a bone marrow cells. A control group was reconstituted with a 50:50 mix of *Roquin*^*+/+*^.Ly5b and *Roquin*^*+/+*^. Ly5a bone marrow. Stem cells present in the bone marrow can reconstitute the hematopoietic compartment of irradiated recipient mice and the congenic markers Ly5a/b allow identification of T cells from Roquin mutant and wild-type origin. miR-146a was upregulated in naive CD4^+^ T cells in a cell-autonomous fashion: only *Roquin*^san/san^ (Ly5b) cells had elevated levels of this miRNA compared with *Roquin*^*+/+*^ (Ly5a) cells in the same mice (Fig. 1e). In the case of miR-21, wild-type cells also had elevated levels of this miRNA compared with the control chimeras (Fig. 1f) suggesting a cell-extrinsic mechanism although a cell-intrinsic component could not be excluded. These results show that Roquin acts in T cells to repress miR-146a and possibly other miRNAs.

### Roquin enhances Dicer-mediated processing of pre-miR-146a

**Figure 2:**
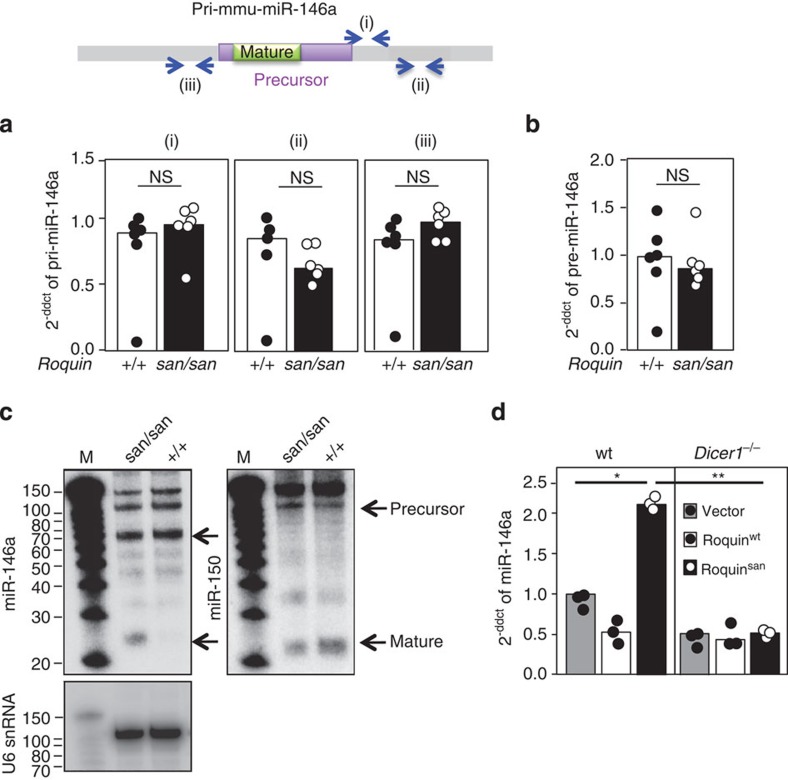
Post-transcriptional Dicer dependent accumulation of miR-146a. (**a**) Relative expression of pri-miR-146a in naive T cells sorted from *Roquin*^*+/+*^ and *Roquin*^san/san^ mice measured by qRT–PCR using three different sets of primers shown as (i), (ii) and (iii) in the schematic. NS, not significant (*P*<0.05, *U*-test). (**b**) The levels of pre-miR-146a in *Roquin*^*+/+*^ and *Roquin*^san/san^ naive T cells measured by qRT–PCR. (**c**) Northern blots showing precursor and mature miRNA transcripts (arrows) of miR-146a and miR-150 in naive T cells of *Roquin*^san/san^ and *Roquin*^*+/+*^ mice. U6 snRNA was used as a loading control and is shown below the miR-146a northern blot. Weaker less abundant bands are miRNA decay products. M, marker. (**d**) Relative expression of miR-146a in *Dicer-floxed-Rosa26-Cre-ER* MEFs (*Dicer1*^*−/−*^) and CD4-CRE (wt) control MEFs assessed by qRT–PCR. MEFs were retrovirally transduced with Roquin^wt^ and Roquin^san^ containing a GFP reporter or with an empty GFP vector control. After 2 days, MEFs were treated with tamoxifen to delete *Dicer.* GFP-positive cells were sorted 7 days later. Each dot represents individual mice (**a**,**b**) or technical replicates (**d**) and the bars represent the median value in each group. *U*-test: *P<0.05, **P<0.005. Results are representative of two independent experiments.

To map the stage at which Roquin represses miR-146a accumulation, we investigated the amount of primary and precursor miRNAs present in naive T cells. qRT–PCR using three different sets of primers to specifically amplify primary miR-146a in naive T cells revealed no differences in the presence of *Roquin*^san^ (Fig. 2a), and this observation extended to primers in the pre-miR-146a region (Fig. 2b). Analysis of miR-146a by northern blotting confirmed the selective accumulation of mature miR-146a in *Roquin*^san/san^ (Fig. 2c), and no accumulation of control miR-150, a miRNA that did not change in *Roquin*^san/san^ T cells. These results point to mature miR-146a accumulating either during or after Dicer-mediated processing.

To investigate Roquin-mediated processing of precursor miRNAs, we next quantified mature miR-146a in MEFs sufficient or deficient in Dicer expression. MEFs were retrovirally transduced with Roquin^wt^, Roquin^san^ or empty vector expressed from an IRES-GFP reporter construct. Twenty-four hours after transduction, all the cells were treated with tamoxifen that removed Dicer from the CreER expressing cells only, and cells were sorted for GFP expression after 1 week. miR-146a accumulation was observed in Dicer-expressing MEFs transduced with Roquin^san^ but accumulation did not occur in the absence of Dicer (Fig. 2d). This indicates that Roquin-induced miR-146a accumulation requires Dicer-mediated processing.

**Figure 3:**
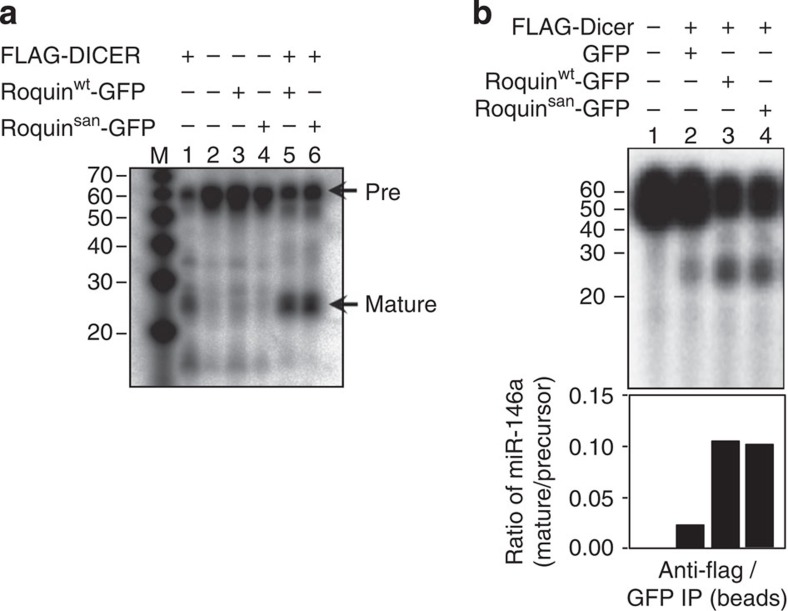
Roquin enhances Dicer mediated pre-miR-146a processing. (**a**) *In vitro* processing of pre-miR-146a in HEK293T cells after transfection with FLAG-Dicer (lane 1), Roquin^wt^-GFP (lane 3) or Roquin^san^-GFP (lane 4). Co-transfection of FLAG-Dicer with either Roquin^wt^-GFP (lane 5) or Roquin^san^-GFP (lane 6) is included. No transfection control is shown in lane 2. (**b**) *In vitro* processing of pre-miR-146a in HEK293T cells after transfection with FLAG-Dicer (lane 2) alone, or co-transfection of FLAG-Dicer with either Roquin^wt^-GFP (lane 3) or Roquin^san^-GFP (lane 4). Lane 1: No transfection control. The lower panels show densitometric analysis of the northern blots to quantify ^32^P. The ratios of the mature versus precursor miRNA intensities are plotted. Intermediates or low abundance compared with the fully digested products were not included in the quantification.

We next investigated whether Roquin itself has miRNA processing activity *in vitro* and can cleave pre-miRNAs into their mature form. Unlike Dicer, Roquin alone did not cleave pre-miR-146a (Fig. 3a). Addition of Roquin enhanced Dicer’s cleavage ability by ~2.5-fold (Fig. 3b) suggesting that it may form a part of the Dicer processing complex. Importantly, the ‘*san’* mutation in Roquin did not further enhance Dicer’s function (Fig. 3b) suggesting that Roquin-induced enhancement of Dicer-mediated processing is not the cause of mature miR-146a accumulation in the presence of Roquin^san^.

### Roquin controls mature miRNA half-life

**Figure 4:**
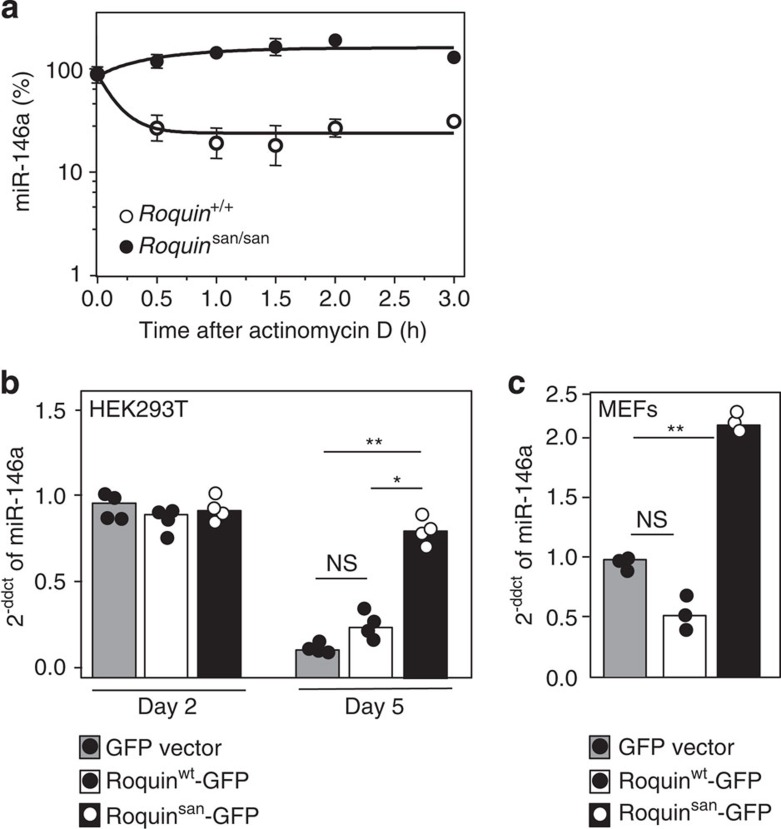
Roquin regulates miR-146a stability *in vitro* and *in vivo*. (**a**) miR-146a in *Roquin*^san/san^ and *Roquin*^*+/*+^ naive T cells measured by qRT–PCR and normalized to comparatively stable U6 snRNA. The cells were treated with actinomycin D (10 mg ml^−1^) for the times indicated. The amount of miR-146a at 0 h was assigned 100%. The error bar represents the range of the data for two experiments. (**b**) Pre-miR-146a was transfected along with Roquin^wt^-GFP or Roquin^san^-GFP in HEK293T cells and qRT–PCR for miR-146a was performed on GFP-positive cells on days 2 and 5 post transfection. See also Supplementary Fig. 2a. (**c**) miR-146a in MEFs 7 days after retroviral transduction of precursor miR-146a along with Roquin^wt^ or Roquin^san^. Each dot represents a technical replicate and the bars represent the median value in each group. *U*-test: *P<0.01, **P<0.001, NS, not significant. The data are representative of two independent experiments.

An explanation for the accumulation of mature miR-146a is increased stability. miR-146a decay was investigated after treatment with actinomycin D^3^ which prevents on-going transcription. The amount of miR-146a dropped below 20% of initial levels within 0.5 h of actinomycin D treatment in wild-type naive CD4^+^ T cells, whereas no decay was observed in *Roquin*^san/san^ cells up to 3 h after treatment, pointing to increased stability as the cause of miR-146a accumulation (Fig. 4a). To study the effects of Roquin on miRNA half-life in isolation from transcription-related events, we transfected HEK293T cells (that express a negligible amount of miR-146a), with synthetic precursor-like miR-146a and either wild-type or mutant Roquin-expressing constructs. Five days post transfection, miR-146a levels had decayed by 50% in the presence of Roquin^wt^, but only by 10% with Roquin^san^ (Fig. 4b). A scramble control miRNA had no effect (Supplementary Fig. 2a). In parallel experiments, retroviruses expressing pre-miR-146a along with retroviral Roquin^wt^ IRES-GFP or Roquin^san^ IRES-GFP were used to transduce wild-type MEFs. GFP^+^ cells were sorted 7 days later and miR-146a was again found to be significantly higher in Roquin^san^ expressing cells (Fig. 4c). Together these results demonstrate that Roquin acts to regulate miRNA decay.

Intracellular localization of miR-146a was investigated by *in situ* hybridization. Cytoplasmic miR-146a^+^ granules were readily visible in *Roquin*^san/san^ naive T cells but not clearly appreciable in control T cells, which precluded comparative quantification. miR-146a did not co-localize with the P-body marker Dcp-1, whereas some miR-146a^+^ granules were eIF3^+^ (Supplementary Fig. 2b) suggesting localization to stress granules. miRNAs are stored in exosomes particularly when found in excess^16^. Consistent with this, there was increased miR-146a within exosomes in *Roquin*^san/san^ T cells in the presence of comparable total numbers of exosomes (Supplementary Fig. 2c–e).

**Figure 5:**
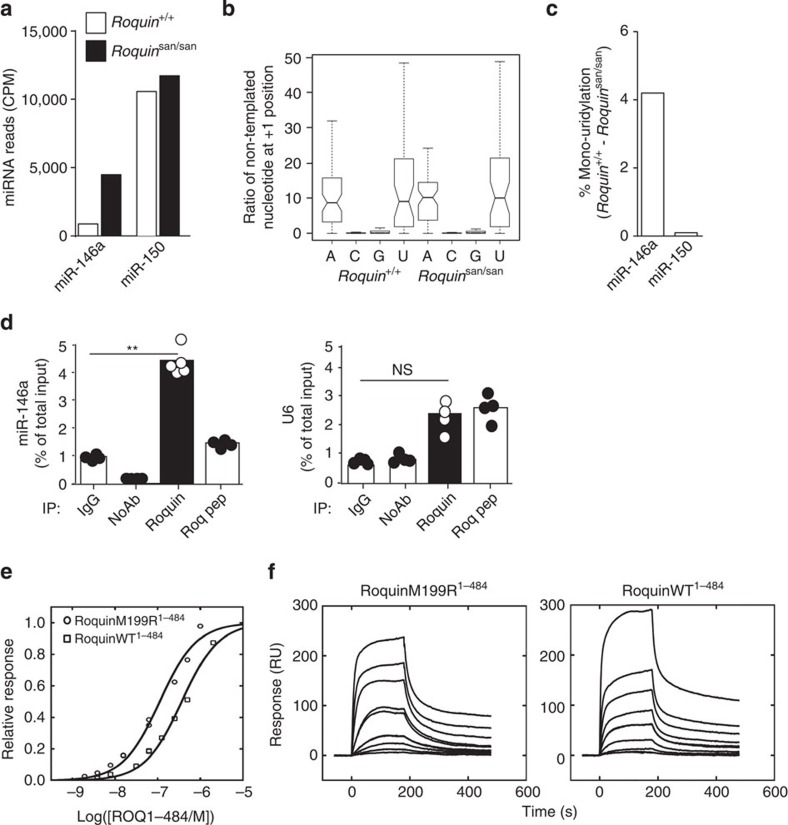
Roquin binds to miR-146a. (**a**) Normalized counts per million (CPM) of miR-146a and miR-150 in sRNA deep-sequencing from *Roquin*^san/san^ versus *Roquin*^*+/+*^ T cells. (**b**) The ratio of non-templated addition of nucleotides at +1 position in *Roquin*^san/san^ and *Roquin*^*+/+*^ T cells. A, adenine; C, cytosine; G, guanine and U, uracil. The two ‘hinges’ are the first and third quartile, the notches extend to ±1.58 × interquartile range/sqrt(no. of observations) and whiskers extend to the data range. (**c**) The percentage of mono-uridylated to exactly matching mature miR-146a or miR-150 was calculated from *Roquin*^*+/+*^ and *Roquin*^san/san^ littermates using the sRNA deep sequencing data set, the percentage change was calculated. sRNA: small RNA. (**d**) qRT–PCR analysis of endogenous miR-146a and U6 in immunoprecipitates from total human tonsil lymphocytes using anti-IgG, no antibody, anti-Roquin IgG (Roquin) and anti-Roquin IgG pre-incubated with a blocking Roquin peptide (Roquin-pep). 10% of the whole cell lysate (total input) was removed before immunoprecipitation and the results are presented as percentage of total input. **P=0.05 (*U*-test), NS, not significant. Each dot represents a technical replicate. The results are representative of five independent experiments. (**e**,**f**) SPR study of the binding of RoquinM199R^1–484^ and RoquinWT^1–484^ to immobilized 5’-biotinylated pre-miR-146a. (**e**) Blank-subtracted Biacore sensograms for RoquinM199R^1–484^ (twofold dilutions, 1,000–3.9 nM) and RoquinWT^1–484^ (2,000–7.8 nM) binding to pre-miR-146a. Protein concentrations increased from the bottom to the top curve. (**f**) Binding isotherms for equilibrium responses. The solid curves were calculated from the derived *K*_D_ values for a 1:1 interaction of 110±20 (for M199R) and 370±50 nM (WT). Fit *R*_max_ values were 330±20 (for WT) and 242±13 response units (M199R).

MiRNA editing and tailing^17^ has been shown to affect miRNA stability in plants^18^ and mammals^19^,^20^. Decreased uridylation has been specifically associated with increased miRNA stability and abundance^19^,^20^,^21^. To investigate possible differences in non-templated additions of miR-146a in the presence of mutant Roquin, small RNA deep sequencing from *Roquin*^*+/+*^ and *Roquin*^san/san^ T cells was carried out. As expected, reads were primarily 22 nt in length (Supplementary Fig. 3a), and differential expression analysis (Supplementary Fig. 3b) confirmed the trends seen with the earlier microarray data (Fig. 1b). Activated *Roquin*^san/san^ CD4^+^ T cells expressed higher levels of miR-146a (Fig. 5a). Overall, non-templated addition was seen largely restricted to A and U and there was no pronounced global change between wild-type and mutant (Fig. 5b). Next, we ranked miRNAs by change in mono-uridylation. Out of 364 analysed miRNAs, miR-146a alongside 39 additional miRNAs were in the top quartile of decreased uridylation (Supplementary Fig. 3c): compared with *Roquin*^*+/+*^ T cells, miRNA-146a uridylation decreased by 4% in *Roquin*^san/san^ (Fig. 5c) T cells. In summary, defective Roquin leads to an increase in miR-146 half-life associated with decreased mono-uridylation and accumulation of the miRNA in exosomes.

### Roquin binds to miR-146a *in vitro* and *in vivo*

MiR-146a binds two *Icos* target sites (TS1 and TS2) that flank the CDE within *Icos* 3′-UTR (Supplementary Fig. 6)^15^. Roquin^7^,^8^ and miR-146a also repress *Icos* mRNA (Pratama *et al.*, submitted). To test whether Roquin can bind miR-146a, we immunoprecipitated Roquin from mouse CD4^+^ T cell lysates and quantified the amount of miR-146a in the precipitate by qRT–PCR. The pull-down using an anti-Roquin polyclonal antibody contained approximately fourfold more miR-146a compared with pull-downs using isotype control or Roquin antibody pre-incubated with a blocking Roquin peptide (Fig. 5d). U6 was amplified as a control; no statistically significant increase specific to Roquin antibody was observed (Fig. 5d). These results show that Roquin forms a complex with mature miR-146a.

To confirm that this was a direct interaction, we used surface plasmon resonance (SPR) experiments with the amino (N)-terminal 1−484 fragment of Roquin (RoquinWT^1–484^) that contains the ROQ and CCCH RNA-binding domains previously shown to bind *ICOS* mRNA by SPR^7^. Both wild-type and mutant Roquin (RoquinM199R^1–484^) were capable of binding miR-146a (Fig. 5e,f) but not miR-21 (data not shown). Binding affinity was approximately threefold higher for mutant Roquin (Fig. 5e,f) as previously observed for *Icos* mRNA^7^.

### The structure of Roquin reveals novel RNA-binding domains

To gain insight into the molecular details of the interaction of Roquin with RNA, we determined the crystal structures of the ROQ domain (residues 177–326, to 2.2 Å) and a larger construct containing the RING, ROQ and zinc-finger domains (residues 1–484, to 2.75 Å) that incorporated the ‘*san*’ mutation M199R (Table 1). There were two copies of the ROQ domain in the asymmetric unit of the first structure, with a dimer interface of 759 Å^2^. Although there was only one molecule in the asymmetric unit of the larger Roquin construct, an identical dimer interface was preserved in the crystal lattice. Although recombinant Roquin protein appeared to be monomeric in solution (data not shown), conservation of the dimer interface in two different crystal forms suggests that it may be biologically relevant (Supplementary Fig. 4a).

**Figure 6:**
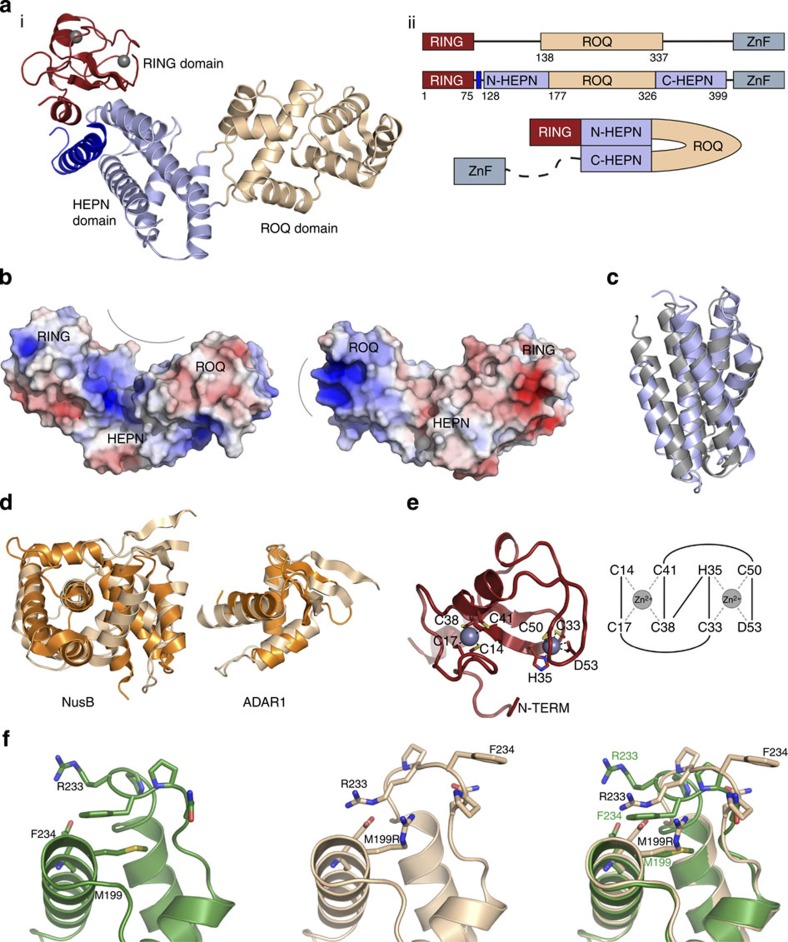
Crystal structure of Roquin fragments. (**a**) Structural and schematic representations of Roquin^1–484^. (i) Overall architecture of Roquin^1–484^ (RING domain=red, ROQ domain=beige, HEPN=light blue, additional helix packing against the HEPN domain=dark blue. (ii) Top: Schematic of the known domain boundaries of Roquin before this work, middle/bottom: domain boundaries revealed by the crystal structure. Note that the ROQ domain is an insertion in the HEPN domain. The ZnF (CCCH) domain was present in the crystallized protein but is not visible in the crystal structure. (**b**) Electrostatic surface diagram of Roquin 1–484 (180 degrees rotated left to right), highlighting positively charged surfaces likely to be involved in RNA binding. The surface indicated in the right hand panel is in a very similar location to the surface used by ADAR1 to bind nucleic acids. The orientation in the left hand panel is identical to that in **a**(i). (**c**) Structural alignment of Roquin HEPN domain with HEPN domain from human Sacsin, (PDB ID:3O10, grey). (**d**) Structural alignment of Roquin ROQ domain (beige) with NusB (PDB ID: 2JR0, orange) and the winged helix-turn-helix from ADAR1 (PDB ID: 1QBJ, orange). (**e**) Structure of the Roquin RING domain and schematic showing the residues involved in coordinating the two zinc atoms. (**f**) Residue F234 is flipped out of the structure in the ROQ M199R mutant, becoming solvent exposed. left: wild-type (green); middle: M199R (beige); right: overlay. See also Supplementary Fig. 4.

The structure of Roquin^1–484^ revealed novel and unexpected domain architecture (Fig. 6a). The RING and ROQ domains could be clearly discerned while there was no electron density associated with the zinc-finger domain, suggesting it is connected to the core of the protein by a flexible linker. Surprisingly, regions of the protein, both N- and carboxy (C)-terminal to the ROQ domain combined to form a HEPN (higher eukaryotes and prokaryotes nucleotide-binding) domain that was not predicted on the basis of sequence. Between the ROQ and HEPN domains is a large concave, positively charged surface reminiscent of nucleic acid-binding sites (Fig. 6b). The HEPN domain consists of a bundle of five α-helices (Fig. 6c, light blue) in an up-down-down-up-down configuration. HEPN domains are ubiquitous in bacteria and have been incorporated into several higher eukaryotic proteins such as human SACSIN (PDB ID 3O10). This was identified as the closest structural homologue using DALI, with root mean squared deviation (r.m.s.d.) of 2.4 Å (Fig. 6c, grey). An additional helix is N-terminal to the HEPN domain, providing extra contact between it and the RING domain.

In Roquin, the 150-residue ROQ domain is inserted between helices 2 and 3 of the HEPN domain (Fig. 6a, beige) and is mostly α-helical. The domain boundaries differed slightly from those suggested by earlier sequence analyses (Fig. 6a). It has seven α-helices interrupted by a short β-strand between helices 2 and 3 and a β-hairpin between helices 4 and 5 (α1-α2-β1-α3-α4-α2-α3-α5-α6-α7). There are no other examples of the overall fold of the ROQ domain in the Protein Data Bank, and therefore this can be considered a new protein fold. The closest structural homologue is the RNA-binding domain of NusB (Fig. 6d, r.m.s.d. 3.3 Å over 88 residues). The ROQ domain can be subdivided into an N-terminal winged helix–turn–helix (α2 to β3), followed by a C-terminal helix–turn–helix (lacking the ‘wing’ of the first, α5 to α7). These were preceded by an extra helix (α1) that appears to stabilize the relative orientation of the two sub-domains. The winged helix–turn–helix (P197–E271) has ~30% sequence identity to DNA-binding Forkhead proteins, and clear structural homology to DNA/RNA-binding proteins including CDT1 (r.m.s.d. 2.8 Å over 83 residues) and ADAR1 (2.6 Å over 62 residues; Fig. 6d).

The RING domain of Roquin adopts the typical zinc-coordinated fold typical of many E3 ubiquitin ligase RING domains. Two atoms of zinc could be clearly discerned in the Roquin structure. One was coordinated by the side-chains of four cysteine residues (C14, C17, C38, C41), while the other was coordinated by two cysteines, a histidine and an aspartate (C33, H35, C50, D53) (Fig. 6e). The presence of an aspartate in the coordination sphere of the second zinc is unusual but not unique^22^.

A comparison of our Roquin^1–484^ structure with that of the wild-type ROQ domain demonstrated that the M199R mutation induces only a minor conformational perturbation but one that is likely to interfere with binding to either an RNA or a protein ligand (Fig. 6f). Although M199 and R199 occupied a similar spatial position, the presence of the mutation (located at the N terminus of α2) resulted in distortion of the C terminus of nearby α3 and the loop that follows it (Fig. 6f, Supplementary Fig. 4b). In particular, E231 and R233 are shifted significantly and F234 flips out from its position between α2 and α3 to become completely solvent exposed. In fact, the conformational change caused by this mutation allows for a crystal contact that is not present in the wild-type protein. RoquinWT^1–484^ did not crystallize under this or any other condition.

### Roquin interacts with the core component of miRISC

**Figure 7:**
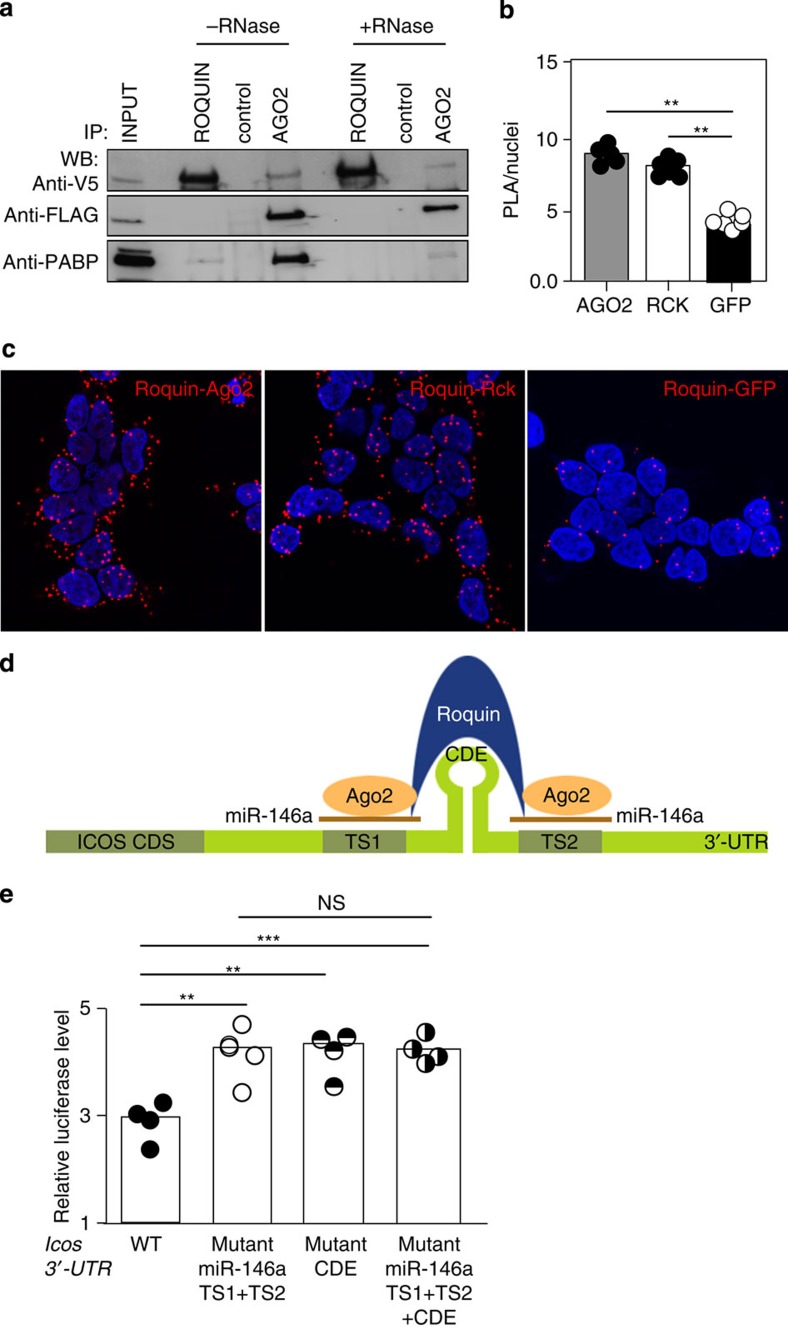
Roquin binds to Ago2. (**a**) Immunoprecipitation of HEK293T cells transiently co-transfected with N-terminal V5-tagged Roquin and C-terminal FLAG-tagged Ago2; using anti-V5 and anti-FLAG antibodies. Half of the immunoprecipitate was treated with RNaseA. Roquin and Ago2 proteins were detected by western blot analysis using anti-V5 and anti-FLAG antibodies. Total lysate is shown in lane 1 as input. Mouse anti-CD71 antibody was used as a control. Anti-PABP antibody was used to confirm the RNase activity. The data are representative of three independent experiments. See also Supplementary Fig. 5. (**b**) Quantification of proximity ligation assays showing average number of PLA spots per average number of cells (PLA/nuclei) for each field of view (represented by the individual dots in each column). At least five fields of view containing 50–100 cells or nuclei were imaged per experiment. Data are representative of four independent assays. *U*-test: **P<0.002. (**c**) Confocal images of HEK293T cells showing molecular proximity of endogenous Roquin and Ago2 (left panel), Roquin and RCK (middle panel) and Roquin and GFP (right panel). Nuclei were stained with DAPI. Scale bar, 25 μm. See also Supplementary Fig. 5. (**d**) Schematic of Roquin binding to miR-146a, Ago2 and *Icos* 3′-UTR containing CDE and miR-146a binding sites TS1 and TS2. See also Supplementary Fig. 6. (**e**) Relative luciferase levels in mouse primary T cells retrovirally transduced with either intact full-length mouse *Icos* 3′-UTR, or this 3′-UTR mRNA carrying mutations in both miR-146a target sites, in the CDE or in both miR-146a target sites plus CDE. Each dot represents a technical replicate. *U*-test: ***P<0.001; **P<0.01. This is a representative of three independent assays.

Roquin binds both miR-146a and its mRNA target (*Icos*), and it shares structural homology with proteins that bind RISC proteins (i.e., ADAR1 (ref. 23)) suggesting that Roquin may itself interact with components of the miRISC complex. We first looked at whether Roquin interacted with Dicer but did not detect an interaction between these proteins when coexpressed in HEK293T (Supplementary Fig. 5a). To investigate a possible interaction between Roquin and the central miRISC component Ago2, V5-Roquin and Ago2-Flag were transfected into 293T cells and the tagged proteins immunoprecipitated. Western blots revealed a weak unidirectional interaction between Roquin and Ago2. This weak one-way interaction was also evident when we immunoprecipitated endogenous Roquin with Ago2 from the murine cell line EL4 (Supplementary Fig. 5b). The interaction was RNA independent as it was also seen in the presence of RNase, which abolished RNA-mediated interaction of PABP1 with Ago2 (ref. 24; Fig. 7a). To confirm that this interaction occurred *in vivo* between endogenously expressed proteins, we used *in situ* proximity ligation assay (PLA), which allows the detection of transient or weak protein interactions. The interaction was probed using mouse anti-Ago2 and rabbit anti-Roquin. Close proximity (<20 nm) allows ligation and amplification of complementary DNA oligonucleotides on secondary antibodies and visualization of individual protein interactions as fluorescent dots. Antibody staining was optimized on endogenous expression of Ago2, Roquin and RCK (Supplementary Fig. 5c) and on exogenous expression of GFP (data not shown). PLA detected interaction between Ago2 and Roquin similar to that of Roquin with its known protein partner RCK^8^ and significantly above background levels seen with GFP (Fig. 7b,c). These results support that Ago2 is a bona fide and close binding partner of Roquin.

Roquin has been shown to bind CDEs within target mRNAs^6^. We compared the consequences of mutating the CDE within the *Icos* 3′-UTR and/or the miR-146a target sites or both (Fig. 7d). Mouse primary T cells were retrovirally transduced with the different *Icos* 3′-UTR mRNA luciferase vectors (Fig. 7e). Mutations in the miR-146a target sites exerted comparable repression defects as the CDE mutation, and there were no additive effects among the different sets of mutations (Fig. 7e). These results show that miR-146a is as effective as Roquin in repressing *Icos* suggesting that they may act in the same pathway, that is, Roquin acts together with miR-146a to exert some or all of the suppression.

## Discussion

Our work identifies a role of the immune modulator Roquin in regulating mammalian miRNA homeostasis by promoting mature miR-146a degradation. T cells expressing Roquin^san^ or lacking Roquin and Roquin-2 displayed comparable accumulation of miR-146a indicating that miRNA dysregulation is due to the loss of the function of both Roquin paralogues. Although 15 miRNAs were elevated at least twofold, miR-146a and miR-21 were the ones most affected. Of these, only miR-146a accumulated in a clearly cell-autonomous manner, suggesting a selective effect of Roquin in the control of miR-146a homeostasis. Some of the other upregulated miRNAs included miR-29b, miR-155 and miR-500, which like miR-146a have been linked either to human or to mouse lupus^25^.

Accumulation of miR-146a in *Roquin*^san/san^ T cells was not found to be a transcriptionally regulated event: neither the primary nor precursor miR-146a levels were different between Roquin mutant and wild-type T cells. Moreover, Roquin does not have a Dicer-like activity that cleaves pre-miRNAs into mature forms. Roquin did enhance miR-146a processing by Dicer but this was not further enhanced with Roquin^san^ excluding this as a possible cause of miR-146a accumulation in Roquin mutant T cells. The presence of mutant Roquin did prolong the half-life of miR-146a, pointing to posttranscriptional stabilization of miR-146a as the cause of the accumulation.

At least two non-mutually exclusive mechanisms may explain miR-146a accumulation in the presence of mutant Roquin: (i) target-mediated microRNA decay (TMMD) and (ii) decreased mono-uridylation. Target mRNAs are not innocently submissive; they can actively regulate the lifespan of the miRNAs that target them. In *C. elegans*, binding of miRNAs to their targets can promote target-mediated microRNA protection^26^, whereas in fly and mammalian cells. the opposite phenomenon—TMMD has been described^27^,^28^,^29^. Given that Roquin binds not only miRNA and target mRNA, but also the RISC component Ago2, it is likely that in the absence of Roquin, a large fraction of cellular miR-146a and target mRNAs are not bound to RISC and are therefore protected from decay by TMMD. Roquin can bind to miR-146a, Ago2 and at least one of miR-146a’s targets, *Icos* mRNA. Roquin may bring miRNAs and their targets into proximity to recruit or help stabilize RISC. Other RNA-binding proteins have been shown to exert the opposite effect, for example, HuR aids miRISC dissociation from its target RNA^30^. Decreased miR-146a mono-uridylation found in the absence of functional Roquin may also contribute to its increased stability: a role for uridylation in enhancing miRNA decay has been described in plants^18^ and mammals^19^,^20^. Intriguingly, HEPN domains are reported in nucleotidyl-transferases^31^. This activity is required for terminal miRNA uridylation; it is therefore tempting to speculate that Roquin may form part of a nucleotidyl–transferase complex to promote uridylation of a subset of miRNAs including miR-146a. Of 39 miRNAs that showed decreased uridylation, only 6 appeared to accumulate in the presence of mutant Roquin. Thus, if decreased uridylation is linked to increased miR-146a stability, this effect is likely to be selective for a small subset of miRNAs, perhaps depending on simultaneous recognition of particular miRNA:mRNA sequences and structures.

Our Roquin structure reveals that the N terminus is composed of RING, HEPN, ROQ and CCCH domains. The structure of the HEPN and ROQ domains suggests that they are highly likely to both function as RNA-binding domains. There are dozens of different classes of RNA-binding domains in eukaryotes^32^ Most RNA-binding proteins contain multiple RNA-binding units to allow for sequence- or structure-specific RNA recognition^33^. In the case of Roquin, we observe at least three different potential RNA-binding domains, the ROQ, HEPN and CCCH domains. The presence of the HEPN domain was unexpected based on sequence analysis as it is discontinuous in the primary sequence as a result of insertion of the ROQ domain between two α-helices that are usually separated in HEPN domains by a variable loop.

To allow for recognition of larger RNA sequences or multiple RNAs, many RNA-binding proteins either dimerize or contain flexible linkers between the individual RNA-binding domains^33^. Roquin appears to use both of these approaches: we see evidence of dimerization as well as the presence of a flexible linker between the HEPN and CCCH domains. One feature common to many RNA-binding domains is the presence of a basic region on their surface that promotes their interaction with the negatively charged nucleic acid backbone. There are two highly positively charged surfaces on Roquin: one is located on the HEPN domain and its interface with the ROQ domain, whereas the other is entirely within the ROQ domain. The latter is the same as the surface used by ADAR1 to bind nucleic acids. ADAR1 complexes with precursor miRNAs and with Dicer through direct protein–protein interaction to also enhance Dicer-mediated pre-miRNA cleavage and facilitate loading of miRNA onto the RISC^23^. Roquin also appears to enhance both Dicer activity and facilitate RISC assembly. This may be related to the significant structural similarity between the N-terminal subdomain of the ROQ domain and the RNA-binding domains of ADAR1: all of them have winged helix–turn–helix structures.

Comparison of wild-type and M199R ROQ domains revealed that the ‘*san*’ mutation causes a structural change in the protein that exposes an otherwise buried hydrophobic residue (F234). Three separate studies in which structures of the ROQ and HEPN domains of Roquin have been solved were published while this paper was under review^34^,^35^,^36^. The two structures presented by Tan *et al.,* are of Roquin bound to RNA CDE motifs from *hmgxb3* and *TNF* mRNAs. These CDEs occupy the two RNA binding sites on the ROQ and HEPN domains we predicted on the basis of electrostatic considerations (Fig. 6b) and the two structures are highly similar to the *apo* structure presented here (r.m.s.d. of 1.9 and 1.1 Å, respectively) indicating that the HEPN and ROQ domains of Roquin undergo only minor structural alterations upon RNA binding. No conclusions regarding the RING domain can be drawn as it is absent in all other structural studies. A comparison of our *san* mutant Roquin with that of RNA-bound wild-type Roquin structures^34^,^35^,^36^ indicates that the *san* mutation does not induce any structural perturbation in either of the RNA binding sites (with the exception of a slight shift in the position of Serine 238). Therefore the severe phenotype seen in the *sanroque* mouse is unlikely to be due to altered RNA binding by Roquin and may instead be due to a failure, by Roquin, to interact with other (protein) components of the mRNA-silencing machinery. This is the subject of future experiments.

Accumulation of miR-146a was more pronounced in naive T cells, known to express genes with longer 3′-UTR than those in proliferating T cells^37^. The components of the miRNA machinery also become less abundant as T cells differentiate into effector cells^38^. In *Dicer*^*−/−*^ MEFs, Roquin regulates *Icos* mRNA independently of miRNAs^8^. The same study also reported that the level of *Icos* repression by Roquin increased with the length of the *Icos* 3′-UTR, indicating that multiple sites in the 3′-UTR may be necessary to induce full repression by Roquin. It is therefore likely that Roquin regulates *Icos* and other target mRNAs via several mechanisms that include microRNA-mediated repression in some cell types.

Transcriptome-wide studies have shown that Roquin controls the degradation of numerous mRNAs with conserved CDE stem-loop motifs^6^. Mutations in the CDE exerted exactly the same inhibitory effect on *Icos* mRNA degradation by Roquin as the mutations in miR-146a target sites, with no additive effect. Thus, it is possible that the suppressive effect of CDE and miR-146a operates through the same pathway. Roquin may bind *Icos* CDE and bring miR-146a to its target sites to facilitate miR-146a repression of *Icos*. Collaboration between AU-rich element-binding microRNAs and AU-rich element-binding proteins is emerging as a prominent mechanism of 3′-UTR-mediated regulation of gene expression in mammalian cells^39^,^40^. As to the question of whether binding of Roquin to the CDE facilitates binding of Roquin to miR-146a, a study published while this paper was under review^34^ confirms that HsRoquin-1 has two binding sites within the ROQ domain. RNA-binding studies showed that the ROQ domain can bind CDEs in one site and dsRNA in the other and that this binding can occur simultaneously and also independently. Combined, these results and ours suggest that binding of Roquin to the CDE may not be needed to facilitate binding of Roquin to miR-146a. Interestingly both RNA-binding sites were shown to be necessary for mRNA decay. Roquin, with its unique structure and different RNA-binding domains may thus be important not only for regulating miRNA homeostasis but also for miRNA function. Further understanding of this Roquin-miR-146a axis may illuminate pathogenesis of inflammatory and autoimmune diseases.

## Methods

### Mice and bone marrow chimeras

C57BL/6(B6) and *Roquin*^san/san^ (*sanroque*) mice were housed in pathogen free conditions at the Australian Phenomics facility at Australian National University (ANU). Seven-to-ten-week-old mice of both genders were used throughout. *Rc3h1*^fl/fl^*;Rc3h2*^fl/fl^; CD4-Cre mice were housed in a pathogen-free barrier facility at the Helmholtz Zentrum, München. For bone marrow chimera reconstitutions, 2 × 10^6^ cells were injected intravenously into 10–12-week-old sublethally irradiated (500 cGy, X-rad) C57BL/6 Ly5a recipients and analysed 12 weeks after reconstitution. The ANU Animal Ethics and Experimentation Committee approved all animal procedures.

### Flow cytometry and microRNA microarray

Single-cell suspensions from spleen and lymph nodes were surface stained for 30 min at 4 °C. Mouse CD4^+^ naive T cells (CD4^+^B220^−^CD44^lo^CD25^−^) were sorted using mouse anti-CD4 1:400 (BioLegend cat#100422 ), anti-CD44 1:200 (BioLegend cat#103006), CD25 1:200 (BioLegend cat#101910) and B220 1:100 (BioLegend cat#103208). For bone marrow chimeras, CD45.1 1:200 (BioLegend cat#110722 )and CD45.2 1:200 (BioLegend cat no#109824) congenic markers were used. Total RNA from T cells from four wt and four mutant mice was extracted using mirVana microRNA isolation kit (Ambion) and miRNA microarrays were performed using Agilent mouse miRNA-v1_95_May07 chips at the Ramaciotti Center for Genomics (UNSW) and analysed using the Genespring software (Agilent Technologies, Inc.)

### qRT–PCR and northern blot

Total RNA was extracted using TRIzol (Life Technologies). Complementary DNA (cDNA) was prepared using miScript RT kit (Qiagen). qRT–PCR for mature and precursor miR-146a, miR-21 and control U6 was performed using the miScript Primer/Precursor Assays (Qiagen) and amplified on an ABI 7900 Prism light cycler in the Biomolecular Resource Facility, ANU. The primer sequences used for detecting primary miR-146a are provided in Supplementary Table 2. The relative expression was calculated using the 2^−ddct^ method^41^.

For measurements of miR-146a in *Rc3h1*^fl/fl^*:/Rc3h2*^fl/fl^;*Cd4-cre*; FACS-sorted naive (CD4^+^CD62L^+^CD44^−^ ) T cells from three *Rc3h1*^fl/fl^*:/Rc3h2*^fl/fl^ (wt) and *Rc3h1*^fl/fl^*:/Rc3h2*^fl/fl^;*Cd4-cre* mice and one *Roquin*^san/san^ mouse were used. miRNA-specific cDNA was prepared using TaqMan MicroRNA Reverse Transcription kit (Applied Biosystems). The expression of miR-146a was measured by qRT–PCR using mmu-miR-146a, snoRNA202 Taqman microRNA Assay (Applied Biosystems) on a Light cycler 480II with the Light cycler 480 SW 1.5 software (Roche).

For northern blot hybridization, 10 μg of total RNA extracted from naive T cells was fractionated on polyacrylamide TBE gel and electroblotted onto Amersham Hybond-N^+^ membrane (GE Healthcare), The miRCURY LNA probes for miR-146a, miR-150 and U6 (Exiqon) were radiolabelled at 5′ end with γ-^32^P ATP and hybridized to the membrane overnight. Radioactive signals were detected with a Typhoon FLA9000 (GE Healthcare).

### Transfections and retroviral transductions

HEK293T cells were transfected with plasmid DNA in six-well plates using Lipofectamine 2000. Roquin^wt^, Roquin^san^ or empty vector expressed from an IRES-GFP. Co-transfections of DNA vectors (4 μg per well) along with a synthetic control or precursor hsa-miR-146a (50 pM, Ambion) was done using Lipofectamine 2000 (Invitrogen) as per the manufacturer’s protocol. The reporter constructs used in transfections and the retroviral transduction method is described previously^2^.

### *In vitro* RNA cleavage assays

*In vitro* RNA cleavage was performed as essentially as described^42^. In brief, synthetic pre-mir-146a oligonucleotides (Integrated DNA Technology) were radiolabelled by 5′ phosphorylation with polynucleotide kinase (NEB) and γ^32^P-ATP. Resulting radiolabelled RNAs were then mixed with anti-Flag (or anti-Flag plus anti-GFP) agarose beads (Sigma) coated with lysates from HEK293T cells transiently transfected with various tagged constructs. Resulting cleavage products were resolved on a urea/acrylamide gel, then visualized and quantified by phosphorimaging.

### Co-immunoprecipitations

Cells were lysed in IP buffer (1% NP-40, 20 mM Tris.HCl (pH=7.4), 150 mM NaCl, 1 × EDTA-free protease inhibitor cocktail (Roche)) for 20 min on ice. The clarified cell lysates were precleared overnight with magnetic Protein G Dynabeads (Invitrogen). The lysates were incubated with 2 μg of antibodies for 1 h and then with Protein G Dynabeads for an additional 3 h at 4 °C before being washed and resuspended in 20 μl of 3X sample buffer (360 mM Tris (pH=6.8), 6% SDS, 30% β-mercaptoethanol, 30% glycerol, 0.15% bromophenol blue). The samples were boiled at 70 °C for 10 mins before use in SDS–polyacrylamide gel electrophoresis(SDS–PAGE). Antibodies used for ectopically expressed constructs were mouse anti-V5 (clone SV5-Pk1, Serotec cat#MCA1360), anti-Flag (clone M2, Sigma cat#F1804), and mouse anti-CD71 (clone MEM-75, Sigma cat# SAB4700515-100UG). Anti-PABP (Abcam, cat#ab21060) was used for assessing RNase activity. For endogenous IPs, mouse anti-Ago2 mAb (Wako cat#014-22023), polyclonal rabbit anti-Roquin (Novus cat#NB100-656) or control mouse anti-CD71 (clone MEM-75, Sigma cat# SAB4700515-100UG) were used..

### *In situ* PLA

PLA was performed according to the manufacturer’s protocol (Duolink kit, Olink Bioscience, Uppsala, Sweden). Briefly, HEK293T cells were grown on coverslips and fixed at room temperature (RT) with 3.7% formaldehyde for 20 min. After three washes with phosphate-buffered saline (PBS), cells were blocked and permeabilized for 60 min in 5% BSA/0.3% Triton-X100 at RT and incubated with primary antibodies at optimized dilutions (rabbit anti-ROQUIN 1:75 (Novus Biologicals); with either mouse anti-RCK 1:150 (Santa Cruz Biotechnology Inc.); mouse anti-Ago2 1:200 (WAKO Pure *Chemical Industries*) or mouse anti-GFP 1:100 (Roche)) overnight in a humid chamber at 4 °C. Slides were washed three times in PBS/0.05% Tween20 and incubated with mouse minus and rabbit plus PLA probes for 1 h at 37 °C. Ligation was carried out for 30 min and amplification for 100 min at 37 °C. Slides were washed, dried at RT in the dark and mounted in mounting media with DAPI (Olink) to stain nuclei. The images were taken on a Leica SP5 confocal microscope with a pin hole of 95.5 μm and a HCxPL APO lambda blue × 631.4 oil objective and quantified using the free software ImageJ (NIH, Maryland, USA).

### RNA immunoprecipitation

For RIP a buffy coat containing lymphocytes was obtained using Ficoll-Paque (GE Healthcare) gradient separation. Total lymphocytes (60 × 10^6^) from tonsils were lysed thoroughly in 1 ml of polysome lysis buffer (100 mM KCl, 5 mM MgCl_2_, 10 mM HEPES, 0.5% NP-40, 1 mM DTT, 0.1 U μl^−1^ RNAse out and 25 μl ml^−1^ protease inhibitor). The total lysate was precleared with 50 μl Protein G Dynabeads (Life Technologies) for 1 h at 4 °C and simultaneously, antibody-bead complex was formed by incubating 15 μl protein G Dynabeads with 3 μg of anti-human Roquin (Bethyl Laboratories *cat#A300–514A*) antibody or control IgG antibody (Santa Cruz cat# sc-2027) for 2 h at 4 °C. A control was included in which the lysate was incubated with Roquin antibody and a blocking peptide. This blocking peptide is the one used for immunization of rabbits to produce the polyclonal anti-Roquin IgG antibody (Bethyl Laboratories, cat #. A300–514A). 10% of the precleared total lysate was saved for use as total input and the rest of the lysate was used for immunoprecipitation. The precleared lysate was combined with antibody–bead complex and incubated at 4 °C for 4 h. After four washes with polysome lysis buffer, the last wash was done with polysome lysis buffer containing 1 M urea for 5 min at 4 °C on a rotating mixer. The beads were then resuspended in 100 μl of polysome lysis buffer containing 0.1% SDS and incubated at 50 °C for 30 min. After incubation, an equal volume of phenol–chloroform was added and RNA was extracted using mirVana miRNA isolation kit (Ambion). RNA was also extracted from the total input. cDNA was prepared using miScript RT kit (Qiagen) and used for qRT–PCR for miRNAs using miScript primers and SYBR Green kit from Qiagen. The *Ct* values were recorded and normalized to the *Ct* values of the total input. The amount of bound miRNA was plotted as percentage enrichment with respective antibody immunoprecipitation.

### Luciferase assays in primary T cells

Wild-type or mutated mouse *Icos* 3′-UTR (miR-146a TS1/2; CDE or combined TS1/2+CDE mutation) DNA fragments were generated with *Xho*I-*Not*I (*GeneArt*; sequence presented in Supplementary Fig. 6) and cloned into retroviral dual-luciferase reporter vector miR-Sens (kind gift from P. Mathijs Voorhoeve). These vectors were then transfected into Phoenix cells, and retroviral supernatants were harvested 48 h post transfection. Primary T cells were retrovirally transduced by spinoculation. The Dual-Glo Luciferase Reporter kit (Promega Corporation) was used to measure luciferase as described previously^43^.

### Small RNA deep sequencing

T cells from spleen and lymph nodes were enriched with an EasySep Mouse T Cell Enrichment Kit (Stemcell, cat# 19751) to >90% purity. RNA was prepared using TRIzol (Life Technologies) and the concentration and integrity of total RNA was verified with an Agilent 2100 Bioanalyser. Small RNA library construction and deep sequencing was done by Illumina HiSeq Technology (Beijing Genomics Institute at Shenzhen). All raw reads were first trimmed to remove the sequencing adaptor (5′-TCGTATGCCGTCTTCTGCTTGT-3′) by using the Trimmomatic software^44^ (parameters: -threads 6 -phred64 input.fq output.t.fq ILLUMINACLIP:adaptor.fa:2:30:10 MINLEN:18) retaining reads that were at least 18 nt long. Then reads were mapped to the mouse reference sequence consisting of the genome sequence (GRCm38), 18s rRNA (gi|374088232) and 28 s rRNA (gi|120444900) using Bowtie (v1.0.1) software^45^. Parameters for mapping were chosen such that three mismatches were allowed in the 18 nt seed region (-n 3 –l 18), the sum of the quality values at the mismatch positions could not exceed 150 (-e 150) and all possible alignments fulfilling these requirements were reported (-a). Other parameters that may influence the alignment output were --nomaqround --maxbts 800 -y --chunkmbs 4096. Alignment files were processed to retain alignments of a read with only minimum mismatches using a Perl script (Supplementary Information 1).

*Expression counts*. All 18–26 nt long sequenced tags were assigned to a mature miRNA if their 5′ start position was within ± 3 nt of the 5′ start position of the mature miRNA (miRBase v21). We call these tags as miRNA mapped tags from here on. Tag counts for mature miRNA were normalized to correct for the library size as follows. If the total tags obtained for a sample is denoted as ‘*N*’, and the raw tags count for a mature miRNA is denoted as ‘*n*’, then the normalized tag count for the mature miRNA refers to (*n* × 1,000,000)/*N*.

*3′ non-templated modifications/additions*. All miRNA-mapped tags that are (a) 1 nt longer than the annotated mature miRNA, and (b) identical to the genome reference sequence except the last nucleotide were considered as tags showing the evidence of 3′ non-templated modifications/additions (Supplementary Fig. 7). The percentage for A,C,G or T non-templated modification/addition was calculated with respect to the sum of mature and all non-templated tags. For example, as indicated in the Supplementary Fig. 7, the percentage for the non-templated T would be *t* × 100/(*m*+*t*+*c*+*g*).

### Protein expression and purification

*Roquin1–484*. GST-Roquin1–484 proteins were expressed in BL21DE3 grown overnight at 18 °C in 0.1 mM zinc acetate and 1 mM IPTG. Pelleted cells were sonicated in R-buffer (20 mM Tris-HCl pH 7.5, 500 mM NaCl, 0.1 mM zinc acetate) with protease inhibitors, RNase A, DNase and lysozyme (Roche). Clarified supernatant was bound to Glutathione Sepharose resin and the tag cleaved with PreScission Protease (GE Healthcare). Eluates were fractionated on a Superdex 200 26/60 column (GE Healthcare). Fractions were analysed by SDS–PAGE and proteins concentrated to 30 mg ml^−1^.

*Roq domain*. The DNA fragment encoding the roq domain (amino acid residues 122–344) was inserted into the expression plasmid pDEST17 by recombination. The expression construct contains C-terminal His tag. The protein was overproduced in *Escherichia coli* strain B834 (DE3). Single colonies were picked and allowed to grow in media containing TB Overnight Express (Novagen) for 5–6 h at 37 °C followed by 20 h at 25 °C. The cell pellet was collected and frozen at 80 °C until use. For protein purification, the cell pellet was resuspended in buffer A (25 mM Tris (pH 7.5), 500 mM NaCl, 30 mM imidazole) with 0.1% Tween 20, protease inhibitors and DNase. The suspension was passed through a cell disruptor at 30 k.p.s.i. and then centrifuged for 30 min at 30,000*g* at 4 °C. The soluble fraction was transferred to an Aktä Express equipped with a Ni-nitrilo-triacetic acid column connected in-line to a Hiload 16/60 Superdex 75 gel filtration column (GE Healthcare). The Ni-nitrilo-triacetic acid column was washed using buffer A, eluted using buffer B (25 mM Tris (pH 7.5), 250 mM NaCl, 250 mM imidazole) before transfer to the gel filtration column equilibrated in buffer C (20 mM Tris (pH 7.5), 200 mM NaCl). Peak fraction collection was performed using the Aktä Express software.

### Surface plasmon resonance (SPR)

RoquinWT^1–484^ and RoquinM199R^1–484^ were freshly dialyzed into SPR buffer (10 mM Tris-HCl pH 7.4, 150 mM NaCl, 1 mM TCEP, 0.05% surfactant P-20). Measurements were made at 20 °C on a Biacore T100 SPR instrument (GE Healthcare) at a flow rate of 50 μl min^−1^. 5′-Biotinylated *mmu-miR-146a* (5′-CUGAGAACUGAAUUCCAUGGGUUAUAUCAAUGUCA-3′) was purchased from Shanghai Gene Pharma Co. Biotinylated. The RNA was dissolved in SPR buffer, heated to 80 °C for 5 min and cooled slowly to allow secondary structure formation. It was immobilized at 20 nM onto a single flow cell of a streptavidin-coated Biacore (SA) chip (GE Healthcare) by injection at 10 μl min^−1^ for ~100 s to get 200 response units. Details of association/dissociation measurements are described in Supplementary Methods. A second flow cell was left underivatized for blank subtraction. Chip surfaces were regenerated by injection of 1 M MgCl_2_ for 1 min at 5 μl min^−1^, leaving the RNA intact while removing bound protein. Association was measured for 180 s and Roquin was allowed to dissociate for 300 s before regeneration of the chip surfaces by injection of 1 M MgCl_2_ for 1 min at 5 μl min^−1^, leaving the RNA intact while removing bound protein. RoquinWT^1–484^ was used in twofold serial dilutions from 2,000–7.8 nM, whereas RoquinM199R^1–484^ was from 1,000–3.9 nM, with injections for each protein done in random order. Although experiments were repeated twice, progressive degradation of the immobilized RNA prevented use of replicates in data fitting. A data set comprising the first 10 cycles measured for each protein was selected to minimize effects from chip degradation.

### Crystallization and structure determination

*Roq domain (Roquin 133–344)*. Crystals of the Roq domain were grown by sitting drops at 20 °C by mixing 10 mg ml^−1^ of the protein (20 mM Tris-HCl pH7.5, 200 mM NaCl) with the condition including lithium chloride 0.2 M and polyethylene 20% Glycol 3350. Crystals were cryo-protected with mother liquor plus 22% glycerol including 0.5 M Kl for 20 s before vitrification in liquid nitrogen. MAD data were collected at 100 K on beamline BM14 at ESRF (Grenoble, France) at wavelengths of 1.55000 (peak) and 0.95350 Å (remote). The diffraction data were processed and scaled with HKL2000 program package^46^. Twenty-one iodine sites in an asymmetric unit were assigned using SHELXD^47^ and used for the SAD phase calculation with SOLVE^48^, resulting in the initial mean figure-of merit of 0.27 for all reflections. We improved the phases using the program DM^49^. Further structure refinement was carried out by PHENIX^50^ using the remote data and manual model building with coot^51^. The final model showed an *R*_free_ factor of 21.4% and an *R* factor of 25.9%. Detailed statistics are summarized in Table 1. Ramachandran Statistics: 97.3% favoured, 2.7% allowed, zero outliers.

*RoquinM199R^1–484^*. Immediately before crystallization, protein was diluted to 10 mg ml^−1^ using 20 mM Tris-HCl pH 7.5, 0.1 mM zinc acetate, to reduce the concentration of NaCl to 167 mM. Crystallization was accomplished by hanging-drop vapour-diffusion at 20 °C using 2 μl drops, and a drop-ratio of 1:1 protein:precipitant. Precipitant was 0.1 M CHES pH 9.5, 7–12% PEG 8000. Crystals grew from phase separation as ~100 μm rhombohedra. Crystals were cryo-protected with mother liquor plus 25% ethylene glycol before vitrification in liquid nitrogen. Zinc-SAD data were collected at 100 K at the Australian Synchrotron on beamline MX2, at a wavelength of 1.265102 Å. Data were integrated using XDS^52^ and scaled using XSCALE. Anomalous signal was present to ~5 Å (6.54–5.35 Å: Anomalous correlation 0.41, SigAno 1.3). Initially a data cut-off of 3.1 Å was used. A molecular replacement solution was found using PHASER, using the ROQ domain (above) as a model. Phases were improved using the anomalous signal from zinc. Two Zn sites were found, and phases were calculated with a mean figure of merit of 0.45 for all reflections. Structure refinement was carried out in PHENIX^50^ using and manual model building with coot^51^. After several rounds of refinement, data were re-processed to include data to 2.75 Å based on a CC_1/2_ >0.1, as suggested by Karplus and Deiderichs^53^, with a significant improvement in map quality. Ramachandran statistics: 98.33% favoured, 1.67% allowed, zero outliers.

### Quantification of secreted exosomes

Splenocytes were stained with B220-FITC, CD3ε-PE and 7AAD. B220-FITC^−^ CD3-PE^hi^/7-AAD^lo^ T cells were sorted. The cells were incubated in anti-CD3ε (BD Pharmingen) pre-coated six-well plates (5 μg ml^−1^) in exosome-depleted complete RPMI medium at 1 × 10^6^ cells ml^−1^ with 2 μg ml^−1^ anti-CD28 antibody (BD Pharmingen) and 20 U ml^−1^ of rIL-2 at 37 °C with 5% CO_2_ for 48 h. After centrifugation for 5 min at 210 g, the supernatant was filtered through a 0.22-μm filter (Merck Millipore) and exosomes harvested by ultracentrifugation at 100,000*g* for 1 h at 4 °C. The exosome pellet was split into two. Half was resuspended in PBS, stained with anti-mouse-CD9-PE antibody (BioLegend) and run on a flow cytometer along the controls (PBS only, PBS plus anti-CD9-PE and unlabelled exosomes). RNA was extracted from the other half of the exosome pellet using TRIzol (Life Technologies). All-in-one miRNA qRT–PCR detection kit (GeneCopoeia) and validated primers for mature miRNAs (GeneCopoeia) were used to quantify mmu-miR-146a-5p and mmu-miR-150-5p. Amplification was detected on a 7900 HT Fast Real-Time PCR system (Applied Biosystems) and relative amounts were calculated using the 2^−dct^.

### *In situ* hybridization

Naive T cells were sorted from *Roquin*^san/san^ and *Roquin*^*+/+*^ mice. The cells were fixed in 3.7% formaldehyde for 20 min at RT. Once fixed, cells were washed once with dH_2_O (13,000 r.p.m. for 10 min) and spotted at 10,000 cells per 25 μl on superfrost plus slides (Thermo Scientific). The slides were incubated at 37 °C. Once the slides were completely dry, they were washed once with 100% ethanol and treated with proteinase K (500 ng ml^−1^) for 5 min at RT. The slides were incubated with the prehybridization solution (Enzo Life Sciences, cat#33808) at 55 °C for 15 min. The cells were hybridized with 2 pM RNA probe at 55 °C in a moist chamber overnight. Alexa-488 conjugated miR-146a LNA probe and Alexa-488 conjugated scrambled LNA probe were purchased from Exiqon. After probe hybridization, cells were blocked with blocking buffer (0.2 × SSC+2% BSA) for 10 min at RT. Primary antibody, Dcp1A (gift from J. Lykke-Andersen, University of California San Diego), and eIf3 were added at a dilution of 1/250 and incubated at RT for 2 h at 4 °C. After three washes with PBST (PBS+0.01% Tween), the slides were incubated with a secondary antibody, donkey-anti-goat-Alexa 568 (Molecular Probes, Invitrogen, cat#A-11057) at RT for 2 h. The cells were washed three times, 10 min each with PBST. Before a final wash with PBST, cells were incubated with DAPI for 2 min. Cells were mounted in Vectashield (Vector Laboratories) and Images were taken using an Olympus IX71 microscope with DP controller software (Olympus) and compiled using Adobe Photoshop software.

## Author contributions

M.S., and G.D. performed most of the experiments and analysed the data. V.A., J.H.C.Y., D.H., S.H.J.B., S.J., A.P. and S.R. helped with the experiments and data analysis. N.J.K., T.O., A.V., E.Y.J. and J.J.B. contributed to the crystal structure of Roquin. H.R.P. and T.P. performed sRNA deep-sequencing analysis. V.A., V.H., M.M.W.C., T.P. and N.E.D. provided intellectual input, expertise and critical reading of the manuscript. M.S. and C.G.V. wrote the manuscript. C.G.V. designed the research and co-supervised the project with V.A.

## Additional information

**Accession codes:** Coordinates and structure factors for Roquin^177–326^ (ROQ domain) and Roquin^1–484^ have been deposited in the Protein Data Bank, accession numbers 3X10 and 4TXA, respectively. All miRNA sequencing data has been submitted to NCBI SRA, accession numbers SRR1724421 and SRR1724420.

**How to cite this article:** Srivastava, M. *et al.* Roquin binds microRNA-146a and Argonaute2 to regulate microRNA homeostasis. *Nat. Commun.* 6:6253 doi: 10.1038/ncomms7253 (2015).

## Supplementary Material

Supplementary Information Supplementary Figures 1-7 and Supplementary Tables 1-2

## Figures and Tables

**Table 1:** Data collection and refinement statistics.

	**RoquinM199R1–484 (Zn SAD)**	**RoquinWT133–344**
*Data collection*		
Space group	C121	C2221
		
*Cell dimensions*		
*a*, *b*, *c* (Å)	142.80, 80.19, 54.86	55.00, 78.32, 184.08
*α*, *β*, *γ* (°)	90.00, 111.06, 90.00	90.00, 90.00, 90.00
Resolution (Å)†	2.75 (2.85–2.75)	2.20 (2.28–2.20)
Rmerge (%)†	11.08 (486)	9.7 (55.2)
*I*/*σ*(*I*)	14.42 (0.52)	17.4 (2.8)
Completeness (%)	98.56 (97.93)	97.4 (90.3)
Redundancy	7.6 (7.7)	8.7 (6.9)
CC1/2†	0.999 (0.16)	(0.862)
		
*Refinement*		
Resolution (Å)†	2.75 (2.90–2.75)	2.20 (2.28–2.20)
No. of unique reflections	14,921 (1,463)	20,060 (2,040)
*R*work/*R*free	21.84/25.53 (41.07/40.02)	21.67/25.21 (22.59/30.00)
		
*No. of atoms*		
Protein	2,836	2,332
Ligands		
Water	0	21
Zinc	2	0
		
*Average B-factors*		
Protein	50.4	39.90
Ligand	68.3	94.90
		
*R.m.s. deviations*		
Bond lengths (Å)	0.004	0.0053
Bond angles (°)	0.87	0.928

Values in parentheses are for highest-resolution shell.

^†^Criteria for data cut-off from ref. 53.
