# Supplementary material for: Roquin binds microRNA-146a and Argonaute2 to regulate microRNA homeostasis
Source: Nat Commun. 2015 Feb 20;6:6253. doi: 10.1038/ncomms7253 (PMC4346627; doi:10.1038/ncomms7253)
Supplement: Supplementary Information — Supplementary Figures 1-7 and Supplementary Tables 1-2 [file ncomms7253-s1.pdf]

Roquin binds microRNA-146a and Argonaute2 to regulate microRNA homeostasis

Monika Srivastava[1], Guowen Duan[1*], Nadia J.Kershaw[2*], Vicki Athanasopoulos[1], Janet H.C. Yeo[3], Toyoyuki Ose[4], Desheng Hu[5], Simon H. J. Brown[6], Slobodan Jergic[6], Hardip. R. Patel[8,9], Alvin Pratama[1], Sashika Richards[1], Anil Verma[4], E. Yvonne Jones[4], Vigo Heissmeyer[5,7], Thomas Preiss[8,10], Nicholas E. Dixon[6], Mark M.W. Chong[3,#], Jeffrey J. Babon[2,#] and Carola G. Vinuesa[1,#]*

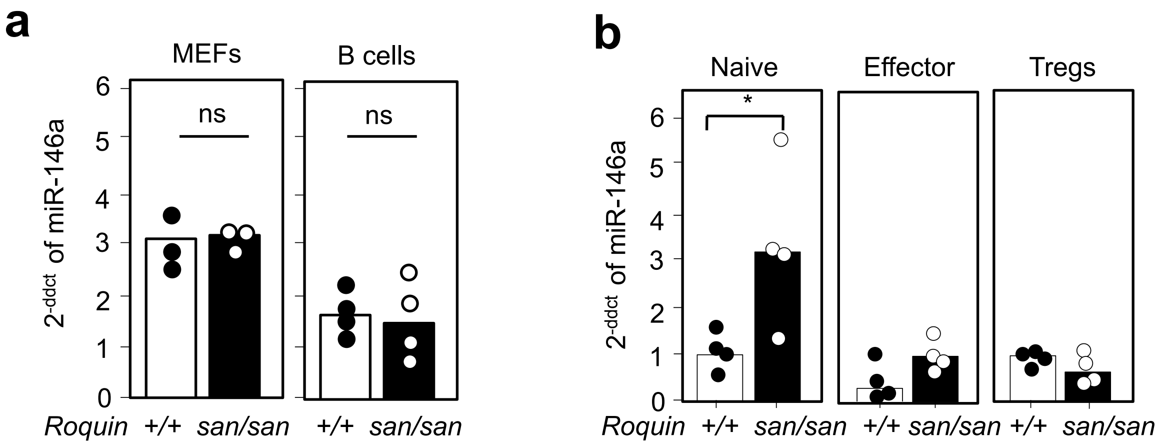

Supplementary Fig. 1. Expression of miR-146a in different cell subsets. Quantitative PCR showing miR-146a expression in *Roquin*$^{san/san}$ and *Roquin*$^{+/+}$ **(a)** MEFs and B cells and **(b)** naïve T cells, effector T cells and regulatory T cells (Tregs). Each dot represents individual mice

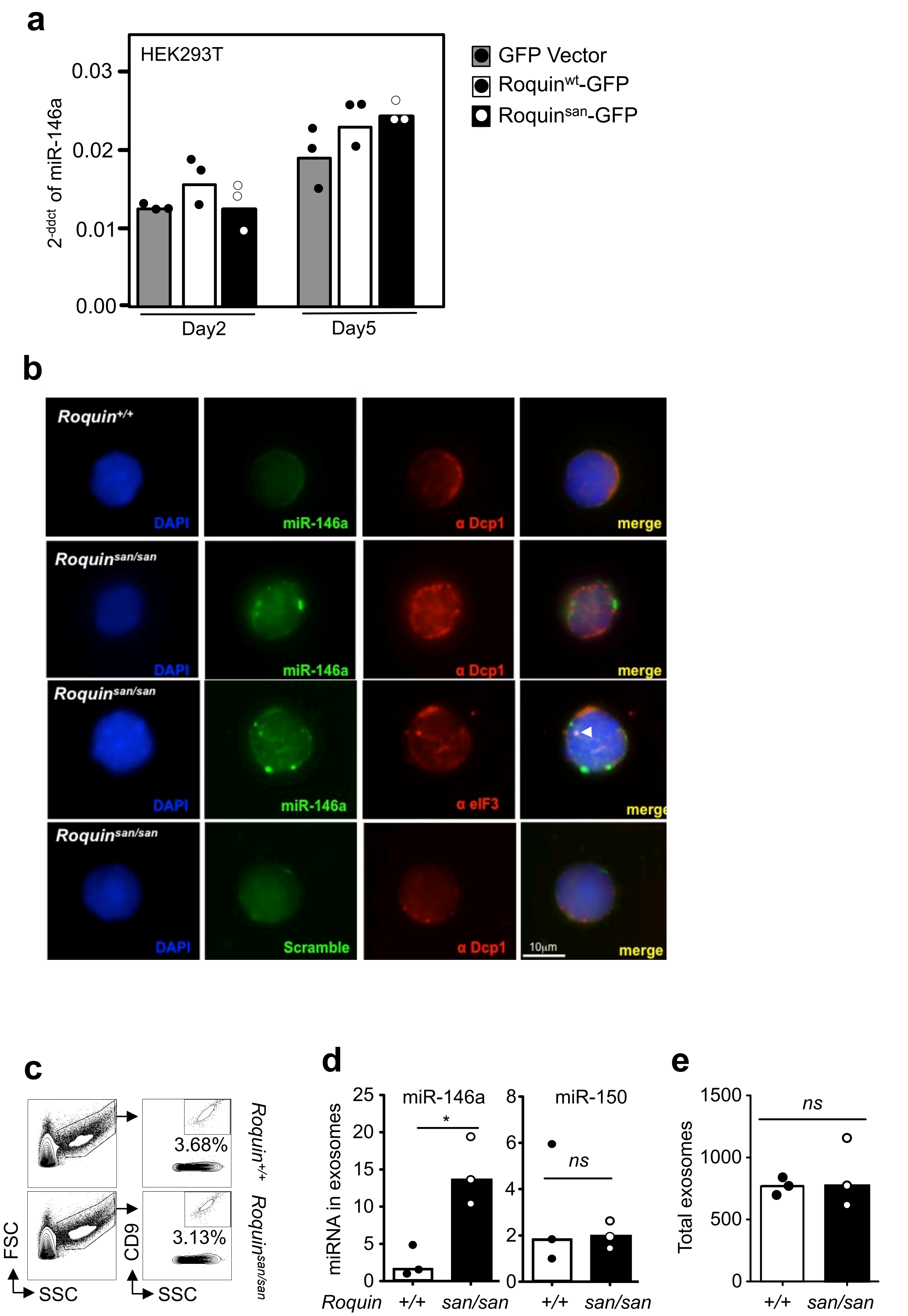

a
HEK293T
0.03
0.02
0.01
0.00
2-ddct of miR-146a
Day2
Day5
GFP Vector
Roquinwt-GFP
Roquinsan-GFP
b
Roquin+/+
Roquinsan/san
DAPI
miR-146a
α Dcp1
α eIF3
Scramble
merge
10µm
c
3.68%
3.13%
FSC
SSC
CD9
Roquin+/+
Roquinsan/san
d
miR-146a
miR-150
miRNA in exosomes
*
ns
Roquin
+/+
san/san
e
Total exosomes
1500
1000
500
0

Supplementary Fig. 2. miR-146a expression, localization and exososome quanitfication.
(a) Cells were co-transfected with scramble miRNA and Roquin^{wt}-GFP or Roquin^{san}-GFP. An empty vector control was also included (lane 1). Cells were sorted for GFP expression at days 2 and 5 and miR-146a was measured by qPCR. Each dot represents a technical replicate and the bar represents the mean in each group.
(b) *In situ* hybridization of miR-146a followed by antibody staining for P body (Dcp1) and stress granule (eIf3) markers in *Roquin$^{san/san}$* naïve T cells. A secondary antibody, anti-goat Alexa-568 (red) was used. A scramble control was also included. Localization of miR-146a with eIf3 in *Roquin$^{san/san}$* (white arrow). Nuclei were stained with DAPI.
(c) Contour FACS plots showing sorting of exosomes from *Roquin$^{+/+}$* and *Roquin$^{san/san}$* T cells. Exosomes were sorted based on CD9 expression.
(d) Quantification of miR-146a and miR-150 in *Roquin$^{+/+}$* an*d Roqui$^{nsan/san}$* T cells.
(e) Quantification of total exosomes in *Roquin$^{+/+}$* and *Roquin$^{san/san}$* T cells. (D-E) Each dot represents an individual mouse and the bar represents the median value in each group. * P=<0.001, ns = not significant.

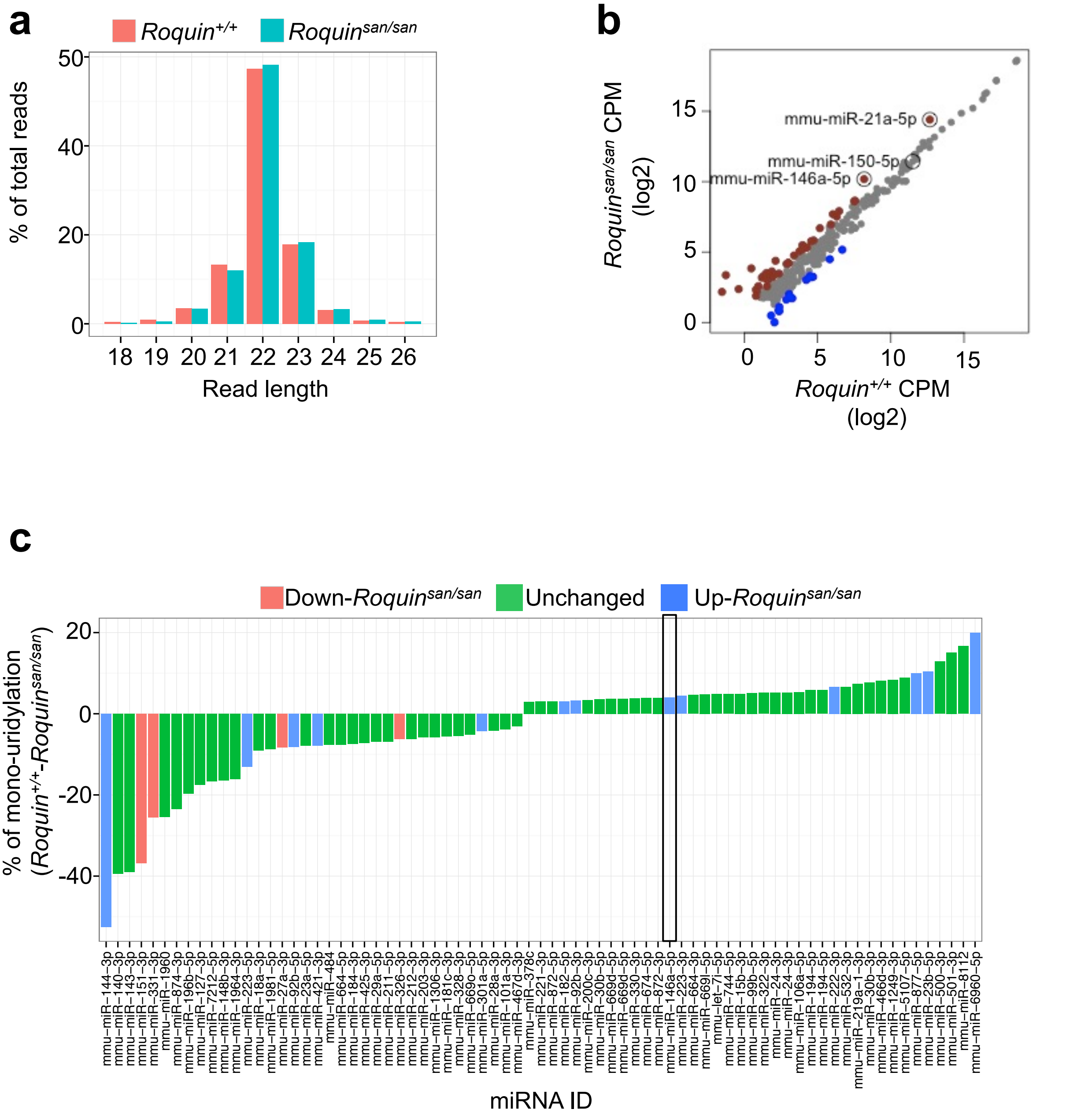

Supplementary Fig.3. sRNA deep sequencing of *Roquin*$^{san/san}$ and *Roquin*$^{+/+}$ T cells.
(a) Percentage of sequences of a given read length in *Roquin*$^{san/san}$ vs *Roquin*$^{+/+}$ T cells sRNA library.
(b) Differential expression of mature miRNAs between *Roquin*$^{san/san}$ vs *Roquin*$^{+/+}$ T cells, expressed as counts per million (CPM) on log2 scale.
(c) Difference in percentage of monouridylation across all miRNAs in *Roquin*$^{san/san}$ vs *Roquin*$^{+/+}$ T cells, selecting the miRNAs in the top and the bottom quartiles. sRNA: small RNA

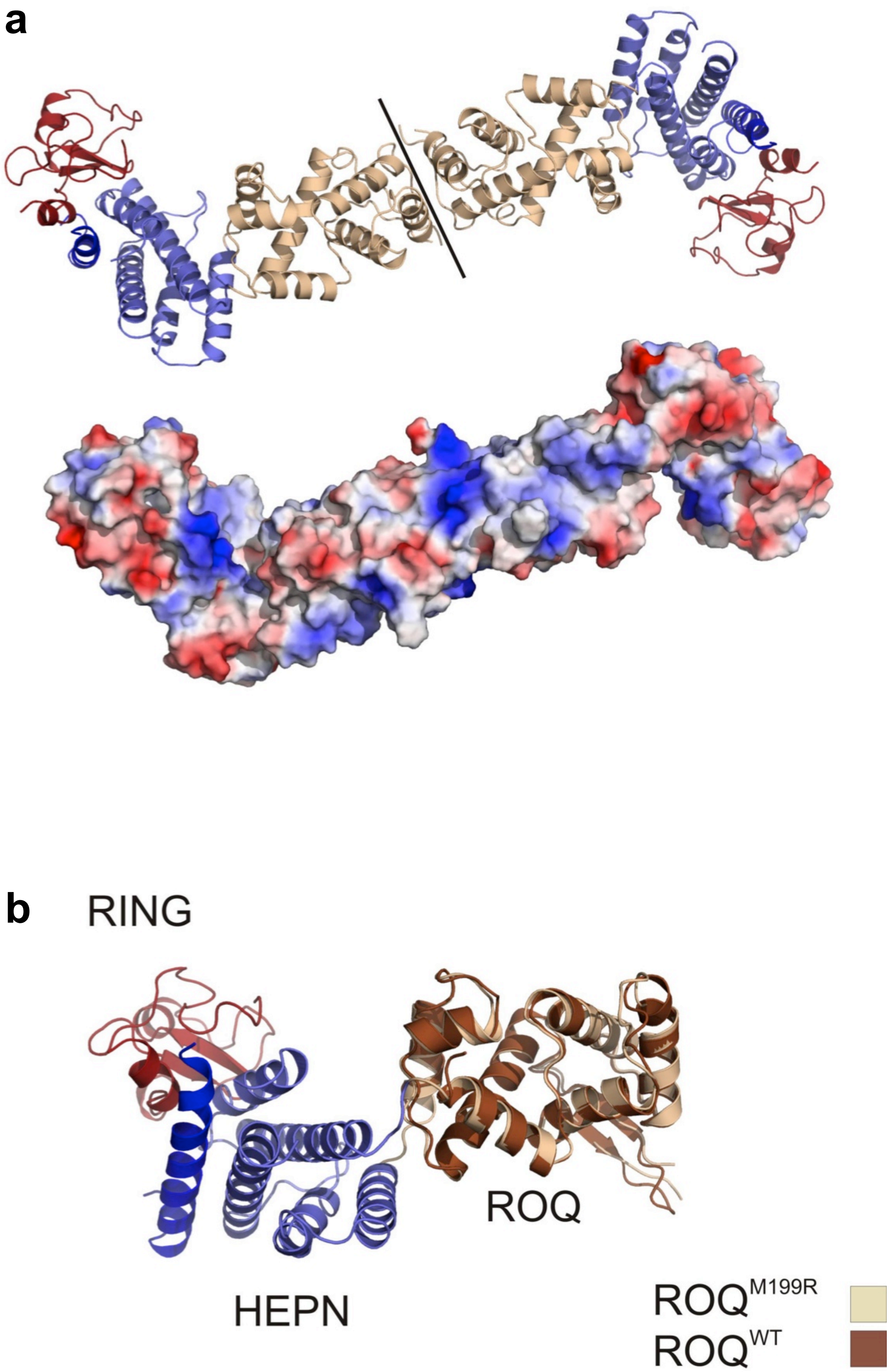

Supplementary Fig. 4. Crystal structure of Roquin.
(a) The Roquin dimer. Ribbon diagram (upper) and electrostatic surface (lower) representation of the Roquin dimer as seen in both crystal structures described in this paper. The dimerization interface is located on the ROQ domain and does not interfere with any of our predicted RNA binding surfaces.
(b) Overlay of the RoquinM199R$^{1\text{-}484}$ and RoquinWT$^{177\text{-}326}$ structures showing that conformational perturbation due to the M199R mutation is limited to a small region that surrounds the site of the mutation.

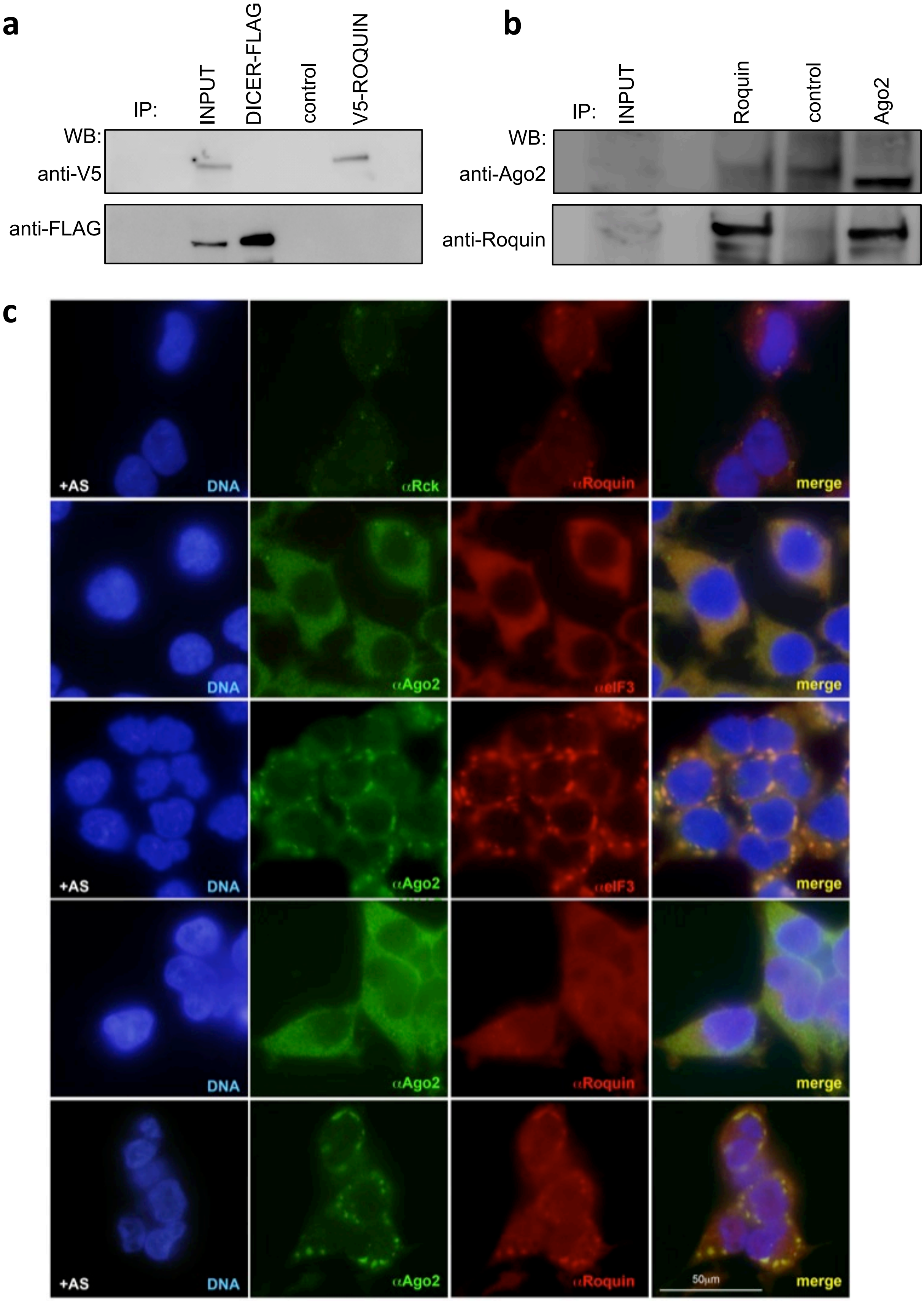

Supplementary Fig. 5. ROQUIN does not interact with DICER.
(a) HEK293T cells were co-transfected with FLAG-DICER and V5-ROQUIN. DICER or ROQUIN were immunoprecipitated using anti-FLAG or anti-V5 antibodies respectively. Total lysate before immunoprecipitation is shown in lane1 (Input).
(b) EL4 cells were lysed and endogenous Ago2 or Roquin were immunoprecipitated with their specific antibodies. Total lysate before immunoprecipitation is shown in lane1 (Input).
(c) Immunofluorescence showing antibody staining of endogenous proteins in the cells used for the PLA assay. Endogenous staining for eIF3 and Roquin is also shown in the presence of arsenite treatment (AS^{+}).

a

MmICOS nt780…2400

…ctcgag

TTTTTCCAGAAAAGACATCTAAATTCAGTTAATATGGTTTACTATGTTGATATTAGTGGTAGTAAACATTTCT
CAGAAACAAAAGCAAATTAATTTTGCTGTGGTGTTTTCTACTATTATCTGTGTTTCCATGGTGCTATTAATCA
CAAGCTTAGCTATTTTTGTAGATCATATTAAAGTTGCAAGCAAGGAAAGCAACCCTCTGTTAATGGGCAAACA
TTCTCCTGGGGTAGAATGAATTATCTATTTAGCCTGAAAGCTGCGGTTTCTGGGTGGTGGCTGCCAGACTACA
GCCACACTTTGCTCTCTCTGGGTTTGACAGGTTGAAGTAGTCCCCCTGGAGCAGCACTCCAGGCTACGCTGGA
GTCCCAGAGTTGGGAGATGCCTGGGACAAGCTGCTGCTTTGGTCTCTGGGATCTGGGAAGTACAGTAGGGCCA
AGACAGAATTCCCTCTCCTAGAAACTATGCAGCCTGGAAGTCAGCCCTAGCACTTTAAGATAGCCTTCTTTAG
AATATGAGTTAGTTGGAAGGATTCTGAAGTGTAAAGAGCATATGACTGCTCTGAGCTGGACCATTTTCTCTAC
TTTCCTGTCTGTATGCCTAAGACTTCTGGAGCAGCCAATGTGTATGCAACATAAAAAAACAAAAAACAAAAAA
ACTCACAAACTTTAAGTAAAATATGGCTGCATCATAGTTTTAAGTTATAGTTGCTTACTTACAGTTTACTTTA
AAAGTGAGCTGTGCATCAGTATATAGTTATTACGTTAAGCATGTGTAATGCTGGCTATGTACAGTACAGTACT
GAACGTGTAATTTGAATCAAGTATGGTGTTCTCTCTCTTCAGATGACCCGGGCAGTCTGACTGGCTTTGTTAG
GGCATTCCCCAAGTTGGACGGTCCTTTCTGTGTGAGGGTGAAGGTGCTTTACTCTGTTCCTTGAAGGATCCCC
ATTCCCAACACGAACACCTAACAATGACTTCTTGAAGAACAAAATTTCTGTGAAATAAAATCTGGCTTAAAGA
GGAGCCACTTGCTAAAAGATGTTAGCAATAATACAAAGTAAAGGCTAAGCACTTGTGTGCAGCAATGGGCTAT
AACCTTGCTGTGTTTTCTGGGGGTGAAGAAGTGATGACCTTAGCTACCATATGCAAATTAGCCCTTGCTCTCC
AATGACCTTTCGTGCTACATAAGATAGTTGCTCCCTTTGTCTCGTTTTTCCCCTTTTAGACTCTGAAATATTC
ACAAGCTTATAAAAGAGTCAGAAGCACAGGAAACAGCACGCTCTGTGCCTGAGTTAGTTTCTGCGTTGACCTC
CTGGCCCCACAGAATGGTGCCTATTTCCTATGGAAATGAAGACTGCACTCTTGATAACTTGACAGTCACCAAT
ATCAGAAAATTAACACTGATATGTCACTACCCTCTAGTCTTTGGTCTGCATTCAAGATCTTCCCCTGGCTTCT
AGGATGTATGTAGGAGCTGTAAGTTCTGCTGGGGATGTCCCTTTGTGTTCGATTGACCTGTCTCGGGAGTGTT
CTTCAGTCTAGACAGTTCTCTTCAGCCTGTAGCCTGGGCCCTTTTGCAGATCACACACCATTATTTTGTAGAA
TGTTCCTTTTGTGGgcggccgc…

b

CDE mutation:

GTATGCGCA

miR-146a target site mutations:

TS1: TGGTGGATCTCCAACAATTCGGA

TS2: TTCTTCAGTCTAGACGATCACTGT

Yellow: miR-146a target sites; green: CDE sequence; Red: mutated nucleotides

Supplementary Fig. 6. miR-146a target sites and CDE within *Icos* 3'UTR.
(a) 3'UTR sequence of *Icos* showing the two binding sites of miR-146a (TS1 and TS2; yellow) and a CDE region (green).
(b) Mutations (red) introduced within the CDE and miR-146a target sites in *Icos* 3'UTR

```
AGCTCTGAGAACTGAATTCCATGGGTTATATCAATGTCAGACCTGTGAAATTCAGTTCTTCAGCT pre-miR-146a
-----TGAGAACTGAATTCCATGGGTT-------------------------------------- mature miR-146a
     TGAGAACTGAATTCCATGGGTTT                                       3' modified (t)
     TGAGAACTGAATTCCATGGGTTC                                       3' modified (c)
     TGAGAACTGAATTCCATGGGTTG                                       3' modified (g)
     TGAGAACTGAATTCCATGGGTTA                                       templated isoform
```

Supplementary Fig. 7. Schematic exemplifying the different putative non-templated (red) vs templated (blue) mono-nucleotides within miR-146a.

	wt1	wt2	wt3	wt4	mean	s/s-1	s/s-2	s/s-3	s/s-4	mean	ratio
mmu-miR-146a	77.6	36.7	64.4	33.6	53.1	3005.2	495.8	1255.5	536.0	1323.1	**24.9**
mmu-miR-21	426.7	112.5	195.2	160.5	223.7	6656.6	2142	2510.7	1112.6	3105.5	**13.9**
mmu-miR-148a	8.8	3.3	2.0	3.5	4.4	58.8	15.4	31.9	8.2	28.6	**6.5**
mmu-miR-500	24.8	13.9	35.9	74.1	37.2	64.5	363.6	61.6	447.3	234.3	**6.3**
mmu-miR-18a	7.5	0.2	1.2	4.3	3.3	27.1	4.7	26.9	1.7	15.1	**4.6**
mmu-miR-451	37.6	23.2	33.6	33.4	32.0	125.4	217.1	198.9	39.7	145.3	**4.5**
mmu-miR-101a	34.4	4.8	8.0	25.9	18.3	100.5	36.8	131.5	21.9	72.7	**4.0**
mmu-miR-222	3.6	2.3	2.6	0.9	2.4	21.7	4.5	6.5	3.3	9.0	**3.8**
mmu-miR-223	30.7	15.8	23.6	15.2	21.3	158	41.5	33.4	59.1	73.0	**3.4**
mmu-miR-155	114.4	68.2	88.4	45.4	79.1	547.6	130.9	219.1	152.4	262.5	**3.3**
mmu-miR-27a	26.7	7.0	10.0	16.1	15.0	106	25.1	42.3	19.7	48.3	**3.2**
mmu-miR-29b	371.8	30.5	50.9	242.1	173.8	466.5	311.2	1229.4	87.2	523.6	**3.0**
mmu-miR-15a	383.9	84.1	95.2	152.1	178.8	798.0	270.1	739.1	189.1	499.1	**2.8**
mmu-miR-24	314.1	109.3	133.7	128.8	171.5	803.7	224.2	502.6	259.6	447.6	**2.6**
mmu-miR-103	164.0	55.4	66.8	56.6	85.7	301.4	73.9	218.4	97.2	172.8	**2.0**

Supplementary Table 1. MicroRNAs upregulated in Roquinsan/san naïve T cells. List of miRNAs upregulated > 2-fold in *Roquin*$^{san/san}$ (s/s) compared to *Roquin*$^{+/+}$ (wt) naïve T cells identified by microRNA microarray, above p=0.01 (ANOVA). The values indicate raw miRNA expression values.

Gene	Primer Sequence
Primary-miR-146a-I	FWD:CTTCAGCTGGGATAGCTCTG, REV:TCCAACCAGTGTTCTTCAGG
Primary-miR-146a-II	FWD:GGCCTTCATAGTCTGCCAAG , REV:CTTCACCCCACTCTCTCCAC
Primary-miR-146a-III	FWD:GGCCGTGCTTTTGGATTTAT , REV:TGCCAGCCCTGTAAAAACAC

Supplementary Table 2. Real time PCR primer sequences for primary miR-146a.
